# Socioeconomic and economic factors affecting access and progression in medical schools: a systematic review and meta-analysis

**DOI:** 10.3352/jeehp.2026.23.6

**Published:** 2026-04-16

**Authors:** Arash Arianpoor, Alexia Pena, Annette Mercer, Jennifer Cox, Dimitra Lekkas, Francis Ruel Geronimo, Heidi Waldron, John Randal, Marcus Dabner, Marita Lynagh, Nalini Pather, Nicole Shepherd, Nigel Robb, Rose Berdin, Tim Wilkinson, Wendy Hu, Boaz Shulruf, Pin-Hsiang Huang

**Affiliations:** 1 School of Clinical Medicine, Faculty of Medicine and Health, The University of New South Wales, Sydney, NSW, Australia; 2 School of Biomedical Sciences, Faculty of Medicine and Health, The University of New South Wales, Sydney, NSW, Australia; 3 School of Medicine, Adelaide University-Robinson Research Institute, Adelaide, SA, Australia; 4 Faculty of Medicine, Nursing & Health Sciences, Monash University, Melbourne, VIC, Australia; 5 School of Dentistry and Medical Science, Charles Sturt University, Orange, NSW, Australia; 6 School of Dentistry, Adelaide University, Adelaide, SA, Australia; 7 School of Rural Medicine, Charles Sturt University, Orange, NSW, Australia; 8 Curtin Medical School, Faculty of Health Sciences, Perth, WA, Australia; 9 Te Herenga Waka-Victoria University of Wellington, Wellington, New Zealand; 10 Medical School, the University of Western Australia, Perth, WA, Australia; 11 School of Medicine & Public Health, University of Newcastle, Newcastle, NSW, Australia; 12 School of Health Sciences, Faculty of Health, Western Sydney University, Sydney, NSW, Australia; 13 Rural Clinical School, University of Tasmania, Burnie, TAS, Australia; 14 School of Medicine and Dentistry, Griffith University, QLD, Australia; 15 Department of Medicine, University of Otago, Christchurch, New Zealand; 16 School of Medicine, Western Sydney University, Sydney, NSW, Australia; 17 Faculty of Medicine and Health, the University of New South Wales, Sydney, NSW, Australia; 18 Centre for Medical and Health Sciences Education, School of Medicine, University of Auckland, Auckland, New Zealand; 19 Department of Medical Humanities and Medical Education, College of Medicine, National Yang Ming Chiao Tung University, Taipei, Taiwan; Hallym University, Korea

**Keywords:** Socioeconomic disparities, School Admission Criteria, Medical education

## Abstract

**Purpose:**

Socioeconomic disadvantage remains a major determinant of equitable access to, and progression within, medical education. This systematic review and meta-analysis examines both the impact and the magnitude of financial and economic disadvantage on student selection and progression in medical school.

**Methods:**

Studies were included if they reported associations between socioeconomic indicators (e.g., parental income, occupation, education, geographic deprivation, or premedical debt) and selection or progression outcomes, and were excluded if they lacked clearly defined economic predictors or sufficient data for binary effect sizes. Searches were conducted across PubMed, Scopus, ERIC, Embase, ProQuest, and EBSCO (2005–2025). Study selection employed an active machine-learning screening process. Extracted data included sample characteristics, socioeconomic measures, and outcome types, with risk of bias assessed using the Risk of Bias Instrument. Random-effects meta-analysis was conducted where appropriate.

**Results:**

Thirty-two studies of medical programs were included, yielding 28 effect sizes for selection and 9 for progression. Household economic and educational disadvantage, identified through parental indices, was consistently associated with reduced odds of admission (odds ratio [OR], 0.6; 95% confidence interval [CI], 0.55–0.65) and poorer progression (OR, 0.56; 95% CI, 0.53–0.59). Geographic deprivation also exerted a negative effect, particularly on selection (OR, 0.69; 95% CI, 0.5–0.93).

**Conclusion:**

Socioeconomic disadvantage exerts a pervasive influence across the medical education continuum. Addressing these inequities requires sustained financial, academic, and psychosocial support both before and during their studies. Students’ economic circumstances should therefore be considered in medical school selection policy and curriculum development to further enhance equity within medical schools and the profession.

## Graphical abstract

[Fig f5-jeehp-23-06]

## Introduction

### Background

Efforts to broaden the diversity of medical school applicants have become an international priority, driven by growing recognition of the need for a health workforce that reflects the full spectrum of socioeconomic diversity in society [1,2]. Despite ongoing equity initiatives, socioeconomic disadvantage remains a major barrier to both entry into and progression through medical programs [3-5]. This continued underrepresentation is concerning, as a more socioeconomically diverse medical workforce is associated with improved health outcomes, enhanced cultural competency, and stronger service provision for underserved communities [1,2,6,7].

Students from lower socioeconomic status (SES) backgrounds continue to face substantial obstacles throughout the medical education continuum [8]. Financial hardship can limit access to preparatory opportunities such as tutoring and admission test preparation, while also intensifying existing family economic pressures. These constraints may influence decisions about pursuing lengthy, costly degrees or taking on debt to fund them [3,5]. Although holistic admissions processes aim to account for applicants’ contextual and life circumstances, such approaches may not fully address entrenched disparities [9]. Moreover, socioeconomic factors appear to continue shaping students’ experiences after admission, affecting academic performance, persistence, and attrition risk [4,10].

Although the problem is widely recognized, limited information is available on the magnitude and persistence of economic influences on both admission and progression outcomes [10]. It remains unclear whether economic background exerts a lasting effect throughout training or whether its influence diminishes over time [10]. While previous studies have broadly explored diversity in medical education participation, there is limited systematic synthesis specifically examining the consequences and magnitude of economic barriers across both student selection and progression.

### Objectives

This systematic review and meta-analysis aimed to address these gaps by synthesizing existing evidence on the influence of financial factors across medical school admission and progression. Specifically, it sought to answer 2 key questions: (1) What are the impacts of both static (e.g., geographic deprivation) and dynamic (e.g., changes in financial status) economic factors on the selection of applicants into medical programs? (2) How do these same factors affect subsequent progression within these programs?

By systematically examining both selection and progression, this review aimed to identify consistent patterns and discrepancies in the literature, thereby informing policies and practices designed to improve equity and socioeconomic diversity in health professions education.

## Methods

### Ethics statement

This systematic review and meta-analysis was based solely on published literature. Therefore, neither institutional review board approval nor informed consent was required.

### Study design

This review followed the Preferred Reporting Items for Systematic Reviews and Meta-Analyses (PRISMA) guidelines [11]. The protocol for this review was not prospectively registered.

### Eligibility criteria

Studies reporting the impact of any individual or composite economic factor on selection or progression outcomes in medical or dental programs were eligible for inclusion. To ensure relevance to the contemporary context, only studies published between January 1, 2005, and December 31, 2025, were considered. Records were excluded if they: (1) did not report original data (e.g., reviews, commentaries, or letters), or (2) were published in languages other than English. Both cross-sectional and longitudinal designs were eligible.

### Information sources and search strategy

A comprehensive, multilayer Boolean search strategy was developed to capture studies at the intersection of 3 domains: (1) economic factors (Layer 1), (2) admission and progression outcomes (Layer 2), and (3) medical and dental programs (Layer 3).

Searches were conducted in July 2025 across Scopus, PubMed, EBSCO, Embase, ERIC, and ProQuest. Following the meta-analysis, a second search using exact Boolean queries was undertaken to identify any newly published studies between the initial search and the time of submission (August 2025–December 2025); none of the newly retrieved records met the inclusion criteria. Overall, the search covered publications from January 1, 2005 to December 31, 2025. A detailed Boolean search strategy is provided in Supplement 1.

### Selection process

After retrieval and deduplication, title and abstract screening was expedited using ASReview Lab (version 2.1.1; release: June 29, 2025; https://asreview.nl/) [12]. All inclusion and exclusion decisions were made and agreed upon by the authors (A.A., B.S., P.H.H.), with consensus sought in cases of ambiguity.

It is important to note that ASReview was used to prioritize records for screening, and screening continued until the predefined stopping rules (SAFE; screen a random set for training data; apply active learning; find more relevant records with a different model; and evaluate quality) were met [13]. Records ranked below the point at which the SAFE criteria were met were not manually screened; instead, they were assumed to be irrelevant based on the model’s predictions and quality checks. The SAFE procedure comprised 5 steps:

#### Step 1

To train the active learning model, a random subset of at least 1% of records from each database was first manually screened. Because the algorithm requires known examples of both inclusion and exclusion criteria to function, this initial training set had to contain at least 1 relevant and 1 irrelevant record [13]. A stratified incremental sampling strategy was used to construct the training set, incorporating the oldest, newest, and randomly selected intermediate records within each database stratum. Inclusion and exclusion decisions were recorded, with the reviewer retaining discretion to screen additional 1% batches until the requirement of identifying at least 1 relevant and 1 irrelevant record was met.

#### Step 2

The labelled training set from Step 1 was imported into ASReview, and titles and abstracts were manually reviewed in the order prioritized by the ELAS-u4 model. The ELAS suite consists of pre-configured artificial intelligence (AI) models; the u-series (Ultra) provides rapid performance frequently used in systematic reviews, while the h-series (Heavy) focuses on deeper semantic understanding of text [14].

#### Step 3

Manual screening within ASReview continued until at least 50 consecutive irrelevant papers were identified. Reviewers could elect to continue if they believed additional relevant records remained. In accordance with established recommendations [13], at least twice the expected number of relevant records in the dataset (*RR_T*) was reviewed before concluding. *RR_T* was calculated as:


(1) $$RR_{-}T=\frac{RR_{-}t}{t}\times T$$

where *RR_t* denotes the number of relevant papers in the training set, *t* the total number of records in the training set, and *T* the total number of records in the full dataset.

#### Step 4

Once all criteria were met, manual title and abstract screening continued, this time using ELAS-h3 (a deep learning model for ranking relevant papers [14]) to minimize the risk of missing relevant records due to model bias.

#### Step 5

Quality assurance was performed by manually reviewing the 10 highest- and lowest-ranked records from the pool of papers that the model predicted to be irrelevant (i.e., those that were excluded from the manual screening phase in Step 3). To further ensure reliability, the entire 5-step procedure was repeated with an independently generated stratified random training set. After title-abstract screening was completed, full texts were retrieved and assessed against the inclusion and exclusion criteria, with disagreements resolved through discussion and consensus.

### Data collection process, data items, effect measures, synthesis methods, and certainty assessment

Full-text review was conducted manually by the primary reviewer (A.A.), who read and contextualized the included papers. All data extraction, including effect sizes and study characteristics, was performed manually, without automated extraction tools. Economic factors reflecting applicants’ or matriculants’ SES were treated as predictors. These included, but were not limited to, geographic deprivation (based on home or school location), parental income, parental occupation, parental education, first-generation status, and composite SES indicators. Outcomes comprised indicators of successful or unsuccessful selection (e.g., admission offer, interview outcome) and progression (e.g., continuation, interruption, graduation, or attrition), provided they were reported as dichotomous outcomes or contained sufficient data to calculate binary effect sizes.

Extracted data included unadjusted odds ratios (ORs) and 95% confidence intervals (CIs). When necessary, raw data were used to calculate or transform ORs. Studies lacking sufficient effect size data were excluded from meta-analysis but summarized narratively.

All data curation and preparation were conducted using Python (https://www.python.org/) in JupyterLab environment (https://github.com/jupyterlab). Data synthesis was subsequently performed in R ver. 4.5.0 (The R Foundation for Statistical Computing) via RStudio (version 1.1.463) using the metafor package ver. 4.8-0 (PBC). Crude ORs extracted from the included studies were natural log-transformed before analysis to ensure mathematical symmetry.

Where at least 2 comparable effect sizes were available across studies for a given predictor-outcome pair, a random-effects meta-analysis was performed. This model was purposefully selected to account for the high between-study variance anticipated from observational data and differing international education systems. Despite this expected heterogeneity, pooling was considered appropriate to synthesize the overarching direction and general magnitude of the associations.

The meta-analytic models used inverse-variance weighting and a restricted maximum likelihood estimator. To avoid double-counting and unit-of-analysis errors when a single study reported multiple effect sizes for the same meta-analytic construct, only the primary reported effect size from that study was used. Finally, statistical heterogeneity across the included studies was quantified using the I^2^ statistic. All R scripts are provided in Supplement 2.

### Risk of bias assessment

To assess potential publication bias, a funnel plot was generated and Egger’s regression test was performed for meta-analyses containing 10 or more studies (k≥10), as tests for asymmetry are insufficiently powered below this threshold. The risk of bias in individual studies was evaluated using the Risk of Bias Instrument developed by the CLARITY Group at McMaster University [15]. Discrepancies were resolved through discussion, and sensitivity analyses were planned on the basis of risk-of-bias ratings.

## Results

### Study selection

**Fig. 1.**
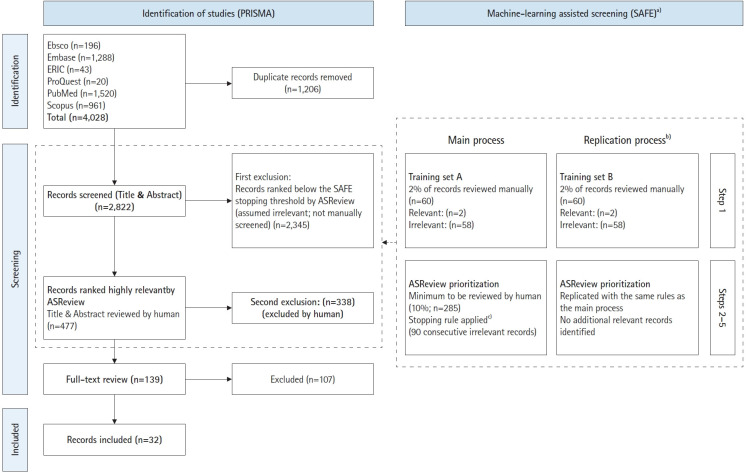
Study selection flowchart. ^a)^Utilizing the ASReview tool, the SAFE procedure was applied for machine learning–assisted title and abstract screening to enhance efficiency and rigor [13]. ^b)^A second random training set was created and the ASReview screening process was replicated to assess reliability; no additional relevant records were identified. ^c)^Based on the training set, the ASReview algorithm using the ultra-fast model (ELAS-u4) prioritized remaining records for review, with a minimum review threshold of 10% (n=285). Screening was stopped after 90 consecutive irrelevant records (stopping rule). Additional review using a deep learning model (ELAS-h3) confirmed stability after 20 further irrelevant records, yielding a total of 477 screened records.

The database search retrieved 4,028 records from Scopus, PubMed, EBSCO, Embase, ERIC, and ProQuest. After deduplication, 2,822 records remained for model training and title-abstract screening (Fig. 1, Supplement 3).

Following the SAFE procedure, an initial random subset of 60 records (approximately 2% of the dataset) was manually screened, identifying 2 relevant and 58 irrelevant papers. This labelled training set was imported into ASReview, and the minimum review threshold was set at 285 records (10% of the dataset) in accordance with the SAFE procedure.

During screening, the reviewer (A.A.) increased the stopping threshold to 90 consecutive irrelevant records when using the ELAS-u4 model and continued with the ELAS-h3 model until an additional 20 consecutive irrelevant records were encountered. In total, 477 titles and abstracts were manually reviewed, with 2,345 records remaining unscreened after the stopping criteria were met; these were considered unlikely to be relevant according to the ASReview ranking. A quality check (Step 5; see Methods) was performed by reviewing the 10 highest- and 10 lowest-ranked unscreened papers, confirming that none were relevant.

To ensure reliability, a second ASReview round using an independent random training set was conducted; no new relevant studies were identified. A relaxed version of the inclusion criteria was used during screening to maximize sensitivity, with potentially relevant records also flagged as relevant. Ultimately, 139 records were reviewed in full text, of which 32 met the inclusion criteria. All included studies examined medical programs, and no studies of dental programs met the eligibility criteria for inclusion (Supplement 4).

### Study characteristics

Included studies provided information on 2 main predictor categories (deprived geographic area and household economic and educational disadvantage) for the selection outcome (Supplement 4), and 3 main categories (deprived geographic area, household economic and educational disadvantage, and premedical debt) for the progression outcome (Supplement 5).

“Household economic and educational disadvantage” was defined as a composite measure combining parental income, occupation, and education where available, with the latter operationalized as first-generation status. It also included inseparable measures (e.g., a score integrating both parental occupation and education) and self-identified measures of disadvantage.

**Fig. 2.**
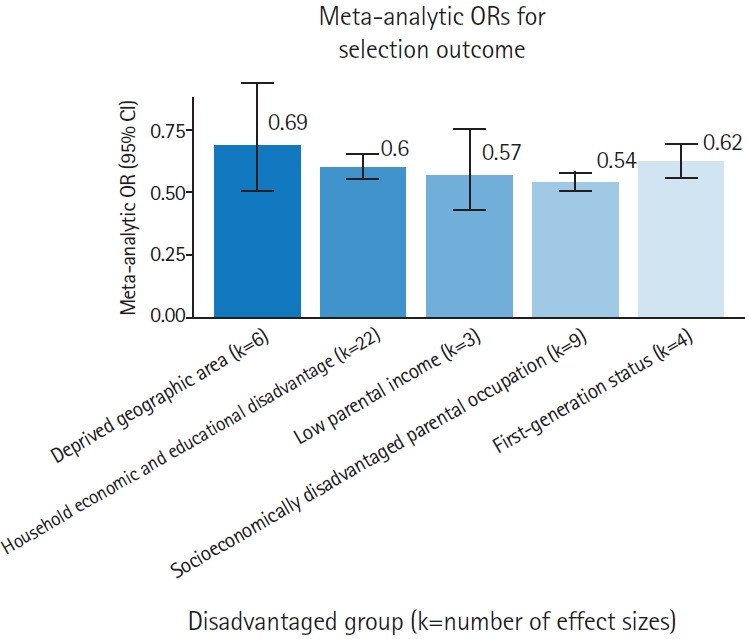
Comparison of effects of socioeconomic predictors on selection outcomes. Lower odds ratios (ORs) indicate greater negative impact of disadvantage. CI, confidence interval.

**Fig. 3.**
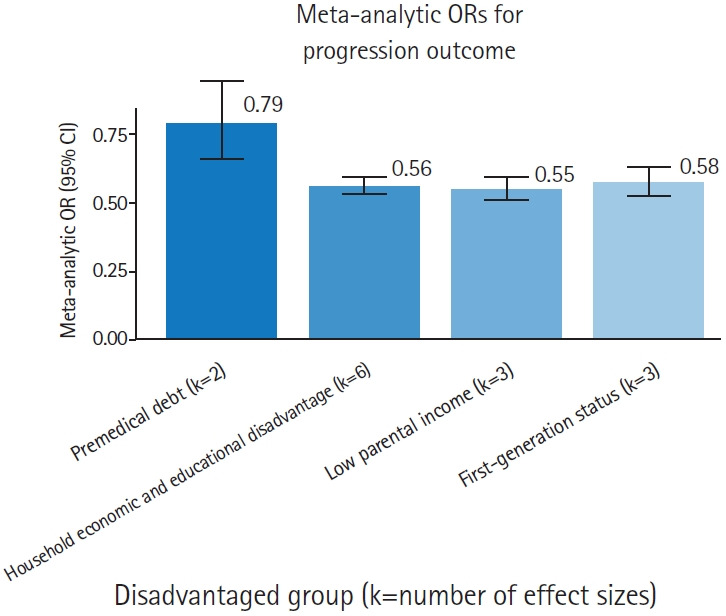
Comparison of effects of socioeconomic predictors on progression outcomes. Lower odds ratios (ORs) indicate greater negative impact of disadvantage. CI, confidence interval.

In total, 28 effect sizes were extracted for the selection outcome (Fig. 2) and 9 for the progression outcome (Fig. 3). It should also be noted that although the review aimed to examine the effects of dynamic economic factors on applicants’ selection and progression, no studies reported extractable effects in this regard.

### Results of the meta-analysis

A summary of the random-effects meta-analyses is presented in Table 1. As anticipated in global educational research, the analyses showed high heterogeneity (I^2^) among the included studies, particularly for selection outcomes, potentially reflecting variation in selection procedures across institutions.

#### Impact of economic factors on student selection

Economic disadvantage was consistently associated with a reduced likelihood of selection into medical programs. The 2 overarching predictor categories (deprived geographic area and household economic and educational disadvantage) both showed negative effects on selection outcomes (Table 1, Fig. 2, Supplement 4).

In particular, applicants from deprived geographic areas, as indicated by neighborhood income or school catchment indices, had a 31% lower likelihood of admission (Table 1, Fig. 2).

For “household economic and educational disadvantage,” meta-analysis of 22 effect sizes showed that being from a disadvantaged household reduced the likelihood of acceptance by 40% (Table 1). Specifically, students from families in the lowest income brackets had an almost 43% lower chance of gaining admission to medical programs (OR, 0.57; 95% CI, 0.43–0.75), those with parents in socioeconomically disadvantaged occupations had a 46% lower likelihood (OR, 0.54; 95% CI, 0.5–0.58), and first-generation university applicants had a 38% lower chance (OR, 0.62; 95% CI, 0.56–0.69).

Overall, applicants from socioeconomically disadvantaged backgrounds were markedly less likely to be admitted to medical programs than their more advantaged peers.

#### Impact of economic factors on student progression

Economic disadvantage was also adversely associated with progression outcomes (Table 1, Fig. 3, Supplement 5).

For “household economic and educational disadvantage,” available data allowed estimation using parental education (first-generation status) and parental income; effect sizes for parental occupation were not available. The pooled composite effect (k=6) revealed that students from economically and educationally disadvantaged households had a 44% lower chance of progressing through medical school (OR, 0.56; 95% CI, 0.53–0.59).

Premedical debt was also negatively associated with progression (OR, 0.79; 95% CI, 0.66–0.94). The effect of deprivation by geographic area was reported in only 1 study, showing a 50% lower chance of progression.

#### Overall impact of socioeconomic disadvantage across the medical education continuum

**Fig. 4.**
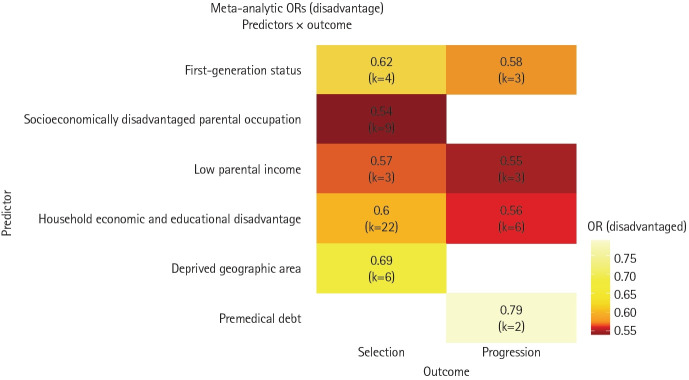
Heatmap of the effects of socioeconomic disadvantage on selection and progression in medical school. In this heatmap, darker colors represent stronger negative effects (lower odds ratios [ORs]), while lighter colors represent weaker negative effects (higher ORs).

As illustrated in Fig. 4, the adverse influence of socioeconomic disadvantage extended beyond the admission stage, consistently affecting both selection and progression outcomes. First-generation applicants and those with parents in the lowest income brackets had reduced odds of both admission (OR, 0.62; 95% CI, 0.56–0.69 and OR, 0.57; 95% CI, 0.43–0.75, respectively) and progression through medical school (OR, 0.58; 95% CI, 0.52–0.63 and OR, 0.55; 95% CI, 0.5–0.59, respectively).

Collectively, these findings highlight a persistent impact of socioeconomic inequality across medical school admission and progression, with disadvantage demonstrating a potentially cumulative effect from selection to completion.

### Risk of bias in studies and reporting biases

Visual inspection of the funnel plot for the composite outcome of household economic and educational disadvantage on selection showed a symmetrical distribution of effect sizes (Supplement 6), and Egger’s test confirmed no significant asymmetry (z=0.075, P=0.940), indicating no statistical evidence of publication bias. Owing to the low number of individual effect sizes pooled in other outcomes, this assessment was not feasible for those specific analyses.

Regarding internal risk of bias, among the 32 included studies, the risk of bias was low for representativeness (21 definitely yes for low risk, 11 probably yes) and missing data (27 definitely yes, 4 probably yes, 1 probably no). Given the nature of this meta-analysis, which aimed to determine the effect of economic factors on selection and progression outcomes, the other 3 bias domains were not applicable, namely response rates, face validity of surveys, and evidence for the reliability and validity of surveys (Supplement 7).

## Discussion

### Interpretation

This systematic review and meta-analysis demonstrates that socioeconomic disadvantage is consistently associated with reduced odds of both admission to and progression through medical school. The negative association is most pronounced for parental and household economic status, whose underlying components correspond to the lowest likelihood of admission and show an even stronger negative relationship with progression once students enter medical training.

#### Disadvantage associated with geographic area

Across diverse jurisdictions, applicants from areas of greater socioeconomic deprivation consistently experience lower rates of enrolment and fewer offers to study medicine [16-19]. For example, the most affluent UK decile receives 23% to 38% of offers, compared with only 1% to 4% among the least affluent [18], with similar associations reported in North America [19,20] and New Zealand [16]. Our meta-analysis supports this disparity, demonstrating 31% lower odds of selection for applicants from deprived geographic areas (OR, 0.69; 95% CI, 0.5–0.93).

When progression is considered, the evidence is more nuanced. Among studies systematically reviewed in the current paper but not included in the meta-analysis, one study from Australia reported that students from very remote areas were more prone to academic difficulty, although these patterns did not always translate into differences in program completion or exit rates [21]. In contrast, a US study found markedly higher attrition among students from under-resourced neighborhoods, with adjusted ORs indicating a 35% increased likelihood of attrition [4] (Supplement 5).

Although the pooled effect indicates a genuine barrier, some studies have reported null or mixed associations [22-25] (Supplements 4, 5). Such inconsistencies may partly reflect differences in institutional admissions policies or methodological limitations, such as reliance on postcode-based SES measures. Geographic areas often encompass considerable socioeconomic heterogeneity, leading to misclassification or ecological fallacy [26]. This can produce perverse equity outcomes, whereby genuinely disadvantaged applicants outside designated low-SES areas are overlooked, while affluent students residing in deprived areas gain unwarranted equity advantages [26-28]. These issues are particularly evident in rural or less urbanized contexts, where individual and neighborhood SES correlate poorly, introducing measurement error [29].

While findings should be interpreted within their local and methodological contexts, the pooled negative effect observed in this meta-analysis may arise from a complex interplay of economic, social, cultural, and educational disadvantages that appear to accumulate across students’ academic trajectories [30,31]. When ecological fallacy is disregarded, geographically disadvantaged students are often assumed to lack the financial capital needed for preparatory materials, as well as the non-financial assets, such as insider knowledge and networks, that affluent peers leverage for elite admissions [32]. Furthermore, community norms and schools in rural or industrial regions often prioritize immediate employment or vocational education and training, inadvertently leaving university-aspiring students feeling isolated or unsupported and viewing higher education as incompatible with community identity [32,33].

Families play a pivotal role in mediating these influences, shaping whether medical training is perceived as attainable. Mitigating these geographical barriers requires strengthening familial and community foundations through outreach, mentoring, and exposure to representative role models [33]. Equally important are policies that create viable educational and professional pathways linking rural origin to return opportunities, such as regional university hubs, rural clinical campuses, and medical programs that emphasize community reinvestment, thereby enabling students to reconcile professional ambition with local belonging.

#### Disadvantage associated with household economic and educational factors

“Household economic and educational disadvantage,” defined here as a composite of low parental income, lower-status occupation, limited parental education, and self-designated disadvantage, was found to have a substantial negative association with selection into medical programs. This disadvantage showed an even more pronounced negative relationship with progression through medical education.

To contextualize these findings, ORs were converted to Cohen’s d, and ratios of progression-to-selection effects were calculated for parental income, first-generation status, and the composite household economic and educational disadvantage. The calculated ratios indicated that the effects of these predictors were meaningfully larger for progression than for selection (Supplement 8).

The general pattern observed suggests that socioeconomic constraints may operate through both financial and sociocultural pathways. Students from low-income households are less likely to apply to highly competitive programs, often perceiving them as exclusive, unattainable, or financially risky [34]. Those who do apply and are accepted may face persistent financial strain that limits engagement and increases stress, often necessitating paid work and contributing to poorer academic performance and higher attrition [30,35,36]. Income-related differences in access to preparatory resources [37] and the influence of parental occupation on early aspirations and professional networking [18,23,33] further compound these disadvantages. Unlike peers from families in higher managerial or professional classes, who benefit from professional role models, students from lower occupational backgrounds often lack the sustained financial, social, and psychological resources critical for navigating medical school [32]. Collectively, these mechanisms help explain why financial and occupational disadvantage may have a stronger impact on progression, where sustained access to financial, social, and psychological resources becomes increasingly important.

Similarly, first-generation university applicant status strongly influences both access and completion rates [5,38-40]. This disadvantage reflects a deeper cultural mismatch between students’ backgrounds and the institutional norms of higher education [41]. Lacking the tacit knowledge transmitted through educated families, such as an understanding of selection tools and academic expectations [34], first-generation students often experience isolation, impostor syndrome, and a hidden curriculum that rewards privileged backgrounds, ultimately undermining their wellbeing and performance [41].

Taken together, these findings highlight that socioeconomic disadvantage constrains both the tangible financial assets and the intangible social and cultural capital needed for success in medical education. Addressing these pervasive inequities requires coordinated institutional change, embedding inclusivity and targeted support within admissions, teaching, and pastoral systems to mitigate the detrimental effects of disadvantage [42].

#### Premedical debt

In our analysis, premedical debt had a generally negative impact on student progression, although this effect was less pronounced than broader indicators of household disadvantage such as parental income or occupation (Table 1, Fig. 3). As this meta-analytic estimate was limited to 2 studies with mixed significance (Supplement 5), these findings warrant cautious interpretation and further empirical research.

Nevertheless, existing literature indicates that high pre-university debt may contribute to increased stress, academic difficulty, and a higher risk of withdrawal or licensure examination failure [43]. This debt-related strain often compels students to take on employment commitments that compromise study time and concentration, thereby increasing the risk of dropout [44].

The precise influence of financial burden on eventual career trajectory remains complex. For instance, recent longitudinal evidence from New Zealand suggests that medical graduates self-report training costs and debt as less important factors influencing their specialty choices, prioritizing working hours and flexibility instead [45]. However, over the longer term, substantial pre-university debt may still subtly shape career trajectories, as structural financial pressures can steer graduates towards higher-paying specialties or occupations to manage repayments [43]. Such ongoing debt aversion limits mobility, delays major life decisions such as home ownership or family formation, and negatively influences overall professional satisfaction [46].

### Comparison with previous studies

No previous meta-analysis has been identified that specifically examines the effects of economic disadvantage on medical or dental student selection and progression. Previous reviews exploring inequity in medical education are largely dated or have focused on comparing selection tools and demographic correlates rather than explicit economic predictors. Furthermore, prior reviews have linked financial hardship to attrition only indirectly, either by proposing theoretical mechanisms without assessing empirical evidence [47] or by noting a severe scarcity of quantitative data regarding social class and parental education [48].

Addressing these gaps, the present meta-analysis provides the first quantitative synthesis demonstrating that economic disadvantage and financial burden remain persistent obstacles to both entry into and progression through medicine. These findings confirm not only the existence of disparities but also the magnitude of their effects, extending earlier qualitative and narrative evidence. Likely mechanisms include heightened financial stress, constrained access to preparatory resources, and reduced academic engagement among students from economically and educationally disadvantaged households.

Future research should move beyond documenting disparities to clarifying causal mechanisms, including how financial pressures interact with institutional cultures, family and community influences, and individual resilience. Emerging evidence also points to both individual- and system-level strategies for widening access [49]. Longitudinal and cross-sectoral approaches are particularly needed to determine whether widening participation initiatives and support programs translate into sustained equity in medical training outcomes and early career progression.

### Limitations

This review and meta-analysis, as a unique synthesis focused specifically on financial disadvantage and selection or progression outcomes, has several limitations.

First, there remains a scarcity of empirical data, particularly studies reporting dichotomized outcomes (selection or progression). Notably, although the search strategy targeted selection and progression in both medical and dental programs, no eligible studies relating to dental programs were identified. This highlights the need for more empirical research on socioeconomic and economic factors affecting access to, and progression within, dental programs, which face challenges similar to those in medical programs. In addition, although the review aimed to examine the effects of dynamic economic factors on applicants’ selection and progression, no studies reported extractable effects in this regard. Although these limitations reduced the number of extractable effect sizes, the study nonetheless provides valuable insights and direction for future research.

Second, the restriction to English-language publications may have excluded relevant studies from non-English-speaking contexts, potentially overlooking differing financial settings. However, this restriction enhances comparability across contexts with broadly similar socioeconomic structures.

Third, medical education systems vary substantially between countries and institutions (for example, publicly funded versus private models, direct-entry versus graduate-entry pathways) and employ diverse widening access initiatives (such as bias minimization strategies, modified selection criteria, community partnerships, and preparatory pathways) [49]. These contextual differences may explain the substantial statistical heterogeneity (I^2^) observed across the included studies, particularly for selection outcomes (Table 1). However, it is important to note that the absolute between-study variance (tau^2^) was consistently low across our analyses, with all but 1 estimate falling well below 0.10. This indicates that although proportional heterogeneity was high, likely driven by the large sample sizes of the primary studies, the actual magnitude of between-study variance in effect sizes was minimal. Consequently, the pooled ORs should be interpreted as robust summary estimates demonstrating a consistent adverse direction of association, rather than as universally applicable effect sizes. Caution is warranted to avoid overinterpreting the exact magnitude of these summary estimates, as the true effect is likely to vary across local institutional and national contexts. Unfortunately, the sparse nature of the extractable data, with few studies per predictor-outcome pair, precluded subgroup or meta-regression analyses to empirically investigate these contextual sources of heterogeneity.

Fourth, the meta-analysis relied on unadjusted ORs to calculate the pooled effects. This approach was necessitated by the inconsistent availability of adjusted ORs (e.g., those controlling for age, gender, or ethnicity) across the included primary studies, which precluded their use in a quantitative synthesis. Consequently, the pooled effect sizes reported in this study may partially reflect the influence of unmeasured confounding variables, and these estimates should be interpreted with appropriate caution.

Fifth, limitations inherent to the use of active machine-learning approaches warrant consideration. Determining the optimal stopping point in active screening requires balancing efficiency against the risk of excluding relevant records. Additional technical challenges include potential model-related omissions [13]. To mitigate these risks, the screening process was extended beyond standard thresholds, repeated with distinct training sets, and supplemented by deep learning models for quality assurance. Moreover, machine learning was used only to prioritize records by predicted relevance; titles and abstracts of highly ranked records were then manually screened by a human reviewer. These steps substantially reduced the likelihood of missing relevant records.

Sixth, although Egger’s test indicated no publication bias for the composite effect of household economic and educational disadvantage on selection, the small number of studies (k<10) in our subgroup analyses precluded formal statistical testing for asymmetry in those specific domains, meaning that the risk of reporting bias in these smaller sub-analyses cannot be entirely ruled out. Moreover, although the CLARITY Group instrument was originally developed for cross-sectional surveys of attitudes and practices, it was selected in this review because it provides a structured and transparent framework for assessing key sources of bias that are broadly relevant to observational studies, including selection processes, measurement validity, and control of confounding. We considered alternative tools for observational research; however, many are tailored to longitudinal or intervention-based designs and may not adequately accommodate the methodological heterogeneity of the included studies. Where specific domains of the CLARITY tool were not applicable, these were explicitly reported rather than scored inappropriately (Supplement 7). In addition, we supplemented the structured assessment with a narrative appraisal of study design and analytical approaches to enhance interpretability. Nevertheless, we acknowledge that use of a tool not specifically designed for all included study designs may limit the precision of the risk-of-bias assessment.

Finally, the protocol for this systematic review was not prospectively registered. Nonetheless, we strictly adhered to PRISMA guidelines throughout the screening and extraction processes to ensure transparency, thereby mitigating the risk of unintentional reporting bias.

### Implications

To date, widening participation initiatives have improved access for underrepresented students [49]. However, our analysis suggests that such policies may be insufficient on their own if they do not also address the ongoing financial and psychosocial pressures that extend from pre-admission through medical training. Equity policies must therefore adopt a longitudinal focus, supporting students from pre-application through graduation rather than treating access and progression as separate challenges.

To mitigate these effects and the disadvantages arising from limited economic resources, policymakers and institutions would need to implement integrated and sustained interventions. These should include financial support to reduce material hardship, alongside wellbeing services, structured mentorship, and learning environments that actively recognize and value socioeconomic diversity. Furthermore, the consistent variation in SES indicators observed across studies underscores the importance of employing multidimensional measures of disadvantage, rather than relying on single proxies such as postcode or parental occupation, to enhance the precision and accountability of future equity policies.

## Conclusion

Socioeconomic disadvantage continues to shape both entry into and progression through medical education, producing persistent inequities that extend beyond initial admission. Our review demonstrates that financial, cultural, and social resources collectively influence not only who is selected but also who thrives and progresses within medical programs.

The key lesson is that achieving genuine inclusivity will require coordinated action across policy, institutional, and community levels, ensuring that medical education fosters not only diversity of entry but also equity of experience and outcomes. In doing so, the profession may move closer to a workforce defined by capability and social representation rather than socioeconomic circumstance.

## Figures and Tables

**Figure f5-jeehp-23-06:**
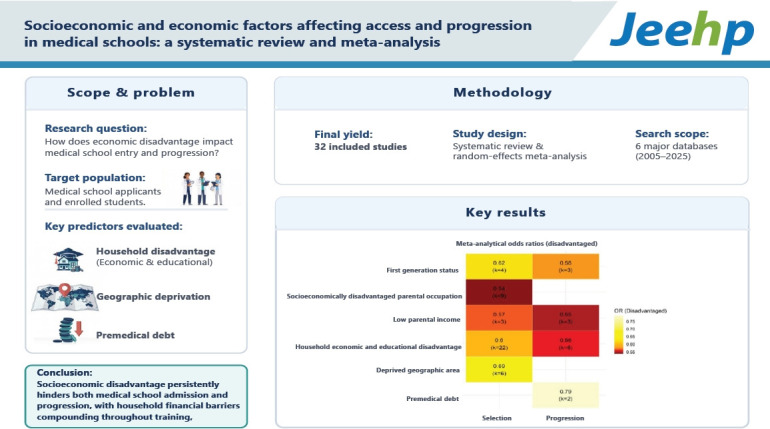


**Table 1.** Meta-analysis of effects of socioeconomic predictors on selection and progression outcomes

Predictor	Selection				Progression			
	No. of effect sizes (K)	Pooled OR (95% CI)	I^2^	τ^2^	No. of effect sizes (K)	Pooled OR (95% CI)	I^2^	τ^2^
Deprived geographic area	6	0.69 (0.5–0.93)	99.28	0.142	1	0.5 (NA)^a)^	NA	NA
Household economic and educational disadvantage								
Composite	22	0.6 (0.55–0.65)	98.68	0.029	6	0.56 (0.53–0.59)	0	0
Parental income	3	0.57 (0.43–0.75)	99.72	0.061	3	0.55 (0.5–0.59)	0	0
Parental occupation	9	0.54 (0.5–0.58)	75.01	0.005	NA	NA	NA	NA
Parental education^b)^	4	0.62 (0.56–0.69)	95.37	0.009	3	0.58 (0.52–0.63)	21.41	0.002
Premedical debt	NA	NA			2	0.79 (0.66–0.94)	25.58	0.008

OR, odds ratio; CI, confidence interval; NA, not available.

^a)^Only one effect size was available. ^b)^Parental education is operationalized as first-generation university applicant/matriculant status.

## Supplementary materials

Supplement 1. Boolean search query.

Supplement 2. R script.

Supplement 3. Record retrieval report.

Supplement 4. Impact of economic factors on students’ selection into medicine and dentistry programs.

Supplement 5. Impact of economic factors on students’ progression in medicine and dentistry programs.

Supplement 6. Funnel plot.

Supplement 7. Bias appraisal.

Supplement 8. Supplementary analysis.

Supplement 9. Audio recording of the abstract.
