# Supplementary material for: Socioeconomic and economic factors affecting access and progression in medical schools: a systematic review and meta-analysis
Source: J Educ Eval Health Prof. 2026 Apr 16;23:6. doi: 10.3352/jeehp.2026.23.6 (PMC13181141; doi:10.3352/jeehp.2026.23.6)
Supplement: Supplementary file 3 — Supplement 1. Boolean search query. [file jeehp-23-06-suppl1.docx]

**Supplement 1.** Boolean search query

This search query was tailored to each database (Scopus, PubMed, EBSCO, Embase, ERIC, and ProQuest) to retrieve relevant records:

TITLE-ABS-KEY ( ( "socioeconomic status" OR "socio-economic status" OR "social determinants" OR "SES" OR "parental education" OR "parental occupation" OR "parental income" OR "family income" OR "social class" OR "deprivation" OR "Gini" OR "disadvantage" OR "upward mobility" OR "downward mobility" OR "social mobility" OR "poverty" OR "affluence" OR "inequality" OR "equity" ) AND ( "admission" OR "selection" OR "offer" OR "entry" OR "acceptance" OR "matriculation" OR "enrolment" OR "enrollment" OR "progression" OR "retention" OR "attrition" OR "dropout" OR "withdrawal" ) AND ( "medical school" OR "dental school" OR "school of medicine" OR "school of dentistry" OR "medical program" OR "dental program" OR "medicine program" OR "dentistry program" ) ) AND PUBYEAR > 2004 AND PUBYEAR < 2026
