# Supplementary material for: Socioeconomic and economic factors affecting access and progression in medical schools: a systematic review and meta-analysis
Source: J Educ Eval Health Prof. 2026 Apr 16;23:6. doi: 10.3352/jeehp.2026.23.6 (PMC13181141; doi:10.3352/jeehp.2026.23.6)
Supplement: Supplementary file 4 — Supplement 2. R script. [file jeehp-23-06-suppl2.docx]

**Supplement 2.** R script

# Read Excel file

data <- read_excel("C:/Users/SAMPLE/Economic factors-Tidy Format-08-10-2025-V8.xlsx")


# Replace 'Not provided' with NA in relevant columns
cols_to_fix <- c('Effect_size', 'Lower_CI', 'Upper_CI')
for (col in cols_to_fix) {
 data[[col]][data[[col]] == 'Not provided'] <- NA
 data[[col]] <- as.numeric(data[[col]])
}


# Keep only rows with complete data for meta-analysis
meta_data <- data[complete.cases(data[, c('Effect_size', 'Lower_CI', 'Upper_CI')]), ]

# Calculate log(OR) and SE for log(OR) on meta_data
meta_data$logOR <- log(meta_data$Effect_size)
meta_data$SE_logOR <- (log(meta_data$Upper_CI) - log(meta_data$Lower_CI)) / (2 * 1.96)

library(writexl)

# Save the cleaned data to a new Excel file
write_xlsx(meta_data, "UCAT_SES_Meta_Cleaned_07_11_2025_V9.xlsx")

# Load the cleaned Excel file
meta_data_clean <- read_excel("UCAT_SES_Meta_Cleaned_07_11_2025_V9.xlsx")

#Meta-analysis

library(metafor)

# Calculate counterpart ORs and CIs on meta_data
meta_data$Counterpart_OR <- 1 / meta_data$Effect_size
meta_data$Counterpart_Lower_CI <- 1 / meta_data$Upper_CI
meta_data$Counterpart_Upper_CI <- 1 / meta_data$Lower_CI

# Calculate log(OR) and SE for counterpart
meta_data$logCounterpart_OR <- -log(meta_data$Effect_size)
meta_data$SE_logCounterpart_OR <- meta_data$SE_logOR # SE remains the same

# Save to Excel
library(writexl)
write_xlsx(meta_data, "UCAT_SES_Meta_Cleaned_07_11_2025_V9_with_Counterparts.xlsx")

# Reload the cleaned Excel file
library(readxl)
data <- read_excel("UCAT_SES_Meta_Cleaned_07_11_2025_V9_with_Counterparts.xlsx")


#Metaanalysis "Debt on Progression"

#Step1
subset_data <- data[
 data$predictor == "Debt" &
 data$outcome == "Progression",
 ]
names(subset_data)

## [1] "Code" "key"
## [3] "author" "title"
## [5] "Field" "cohort"
## [7] "Date_range" "predictor"
## [9] "predictor_indicator" "outcome"
## [11] "outcome_indicator" "Effect_direction"
## [13] "Effect_Description" "Effect_type (Cohen's d, OR, etc)"
## [15] "Effect_size" "Lower_CI"
## [17] "Upper_CI" "n_applicant"
## [19] "n_selected" "n_enrollee"
## [21] "n_progressed" "logOR"
## [23] "SE_logOR" "Counterpart_OR"
## [25] "Counterpart_Lower_CI" "Counterpart_Upper_CI"
## [27] "logCounterpart_OR" "SE_logCounterpart_OR"

##Step2: "Having Debt"
library(metafor)
res_OR <- rma(
 yi = subset_data$logOR,
 sei = subset_data$SE_logOR,
 method = "REML",
 slab = subset_data$author
)
summary(res_OR)

##
## Random-Effects Model (k = 2; tau^2 estimator: REML)
##
## logLik deviance AIC BIC AICc
## 0.2971 -0.5943 3.4057 -0.5943 15.4057
##
## tau^2 (estimated amount of total heterogeneity): 0.0083 (SE = 0.0457)
## tau (square root of estimated tau^2 value): 0.0909
## I^2 (total heterogeneity / total variability): 25.58%
## H^2 (total variability / sampling variability): 1.34
##
## Test for Heterogeneity:
## Q(df = 1) = 1.3437, p-val = 0.2464
##
## Model Results:
##
## estimate se zval pval ci.lb ci.ub
## -0.2355 0.0915 -2.5727 0.0101 -0.4149 -0.0561 *
##
## ---
## Signif. codes: 0 '***' 0.001 '**' 0.01 '*' 0.05 '.' 0.1 ' ' 1

###Step3: "Not Having Debt"
res_Counterpart <- rma(
 yi = subset_data$logCounterpart_OR,
 sei = subset_data$SE_logCounterpart_OR,
 method = "REML",
 slab = subset_data$author
)
summary(res_Counterpart)

##
## Random-Effects Model (k = 2; tau^2 estimator: REML)
##
## logLik deviance AIC BIC AICc
## 0.2971 -0.5943 3.4057 -0.5943 15.4057
##
## tau^2 (estimated amount of total heterogeneity): 0.0083 (SE = 0.0457)
## tau (square root of estimated tau^2 value): 0.0909
## I^2 (total heterogeneity / total variability): 25.58%
## H^2 (total variability / sampling variability): 1.34
##
## Test for Heterogeneity:
## Q(df = 1) = 1.3437, p-val = 0.2464
##
## Model Results:
##
## estimate se zval pval ci.lb ci.ub
## 0.2355 0.0915 2.5727 0.0101 0.0561 0.4149 *
##
## ---
## Signif. codes: 0 '***' 0.001 '**' 0.01 '*' 0.05 '.' 0.1 ' ' 1

###Step4:
library(writexl)

# Helper function to extract meta-analysis summary
extract_ma_results <- function(res, label) {
 data.frame(
 Effect = label,
 Model = res$method, # e.g., REML
 Studies = res$k,
 Estimate = exp(res$b),
 Lower_CI = exp(res$ci.lb),
 Upper_CI = exp(res$ci.ub),
 logOR = res$b,
 SE_logOR = res$se,
 z_value = res$zval,
 p_value = res$pval,
 Q = res$QE,
 Q_p = res$QEp,
 I2 = res$I2,
 tau2 = res$tau2,
 Pred_Lower = if(!is.null(res$pi.lb)) exp(res$pi.lb) else NA,
 Pred_Upper = if(!is.null(res$pi.ub)) exp(res$pi.ub) else NA
 )
}

results <- rbind(
 extract_ma_results(res_OR, "Debt"),
 extract_ma_results(res_Counterpart, "Debt (Counterpart)")
)

write_xlsx(results, "MA_Debt_Progression_07_11_2025.xlsx")


#Metaanalysis "Socioeconomic/Educational Disadvantage Composite on Progression"

#Step1
subset_data <- data[
 data$predictor == "Socioeconomic/Educational Disadvantage Composite" &
 data$outcome == "Progression",
 ]
names(subset_data)

## [1] "Code" "key"
## [3] "author" "title"
## [5] "Field" "cohort"
## [7] "Date_range" "predictor"
## [9] "predictor_indicator" "outcome"
## [11] "outcome_indicator" "Effect_direction"
## [13] "Effect_Description" "Effect_type (Cohen's d, OR, etc)"
## [15] "Effect_size" "Lower_CI"
## [17] "Upper_CI" "n_applicant"
## [19] "n_selected" "n_enrollee"
## [21] "n_progressed" "logOR"
## [23] "SE_logOR" "Counterpart_OR"
## [25] "Counterpart_Lower_CI" "Counterpart_Upper_CI"
## [27] "logCounterpart_OR" "SE_logCounterpart_OR"

##Step2: "Being from a low income family"
library(metafor)
res_OR <- rma(
 yi = subset_data$logOR,
 sei = subset_data$SE_logOR,
 method = "REML",
 slab = subset_data$author
)
summary(res_OR)

##
## Random-Effects Model (k = 6; tau^2 estimator: REML)
##
## logLik deviance AIC BIC AICc
## 5.3800 -10.7601 -6.7601 -7.5412 -0.7601
##
## tau^2 (estimated amount of total heterogeneity): 0 (SE = 0.0025)
## tau (square root of estimated tau^2 value): 0
## I^2 (total heterogeneity / total variability): 0.00%
## H^2 (total variability / sampling variability): 1.00
##
## Test for Heterogeneity:
## Q(df = 5) = 2.3482, p-val = 0.7992
##
## Model Results:
##
## estimate se zval pval ci.lb ci.ub
## -0.5778 0.0274 -21.1256 <.0001 -0.6314 -0.5242 ***
##
## ---
## Signif. codes: 0 '***' 0.001 '**' 0.01 '*' 0.05 '.' 0.1 ' ' 1

###Step3: "Not being from a low income family"
res_Counterpart <- rma(
 yi = subset_data$logCounterpart_OR,
 sei = subset_data$SE_logCounterpart_OR,
 method = "REML",
 slab = subset_data$author
)
summary(res_Counterpart)

##
## Random-Effects Model (k = 6; tau^2 estimator: REML)
##
## logLik deviance AIC BIC AICc
## 5.3800 -10.7601 -6.7601 -7.5412 -0.7601
##
## tau^2 (estimated amount of total heterogeneity): 0 (SE = 0.0025)
## tau (square root of estimated tau^2 value): 0
## I^2 (total heterogeneity / total variability): 0.00%
## H^2 (total variability / sampling variability): 1.00
##
## Test for Heterogeneity:
## Q(df = 5) = 2.3482, p-val = 0.7992
##
## Model Results:
##
## estimate se zval pval ci.lb ci.ub
## 0.5778 0.0274 21.1256 <.0001 0.5242 0.6314 ***
##
## ---
## Signif. codes: 0 '***' 0.001 '**' 0.01 '*' 0.05 '.' 0.1 ' ' 1

###Step4:
library(writexl)

# Helper function to extract meta-analysis summary
extract_ma_results <- function(res, label) {
 data.frame(
 Effect = label,
 Model = res$method, # e.g., REML
 Studies = res$k,
 Estimate = exp(res$b),
 Lower_CI = exp(res$ci.lb),
 Upper_CI = exp(res$ci.ub),
 logOR = res$b,
 SE_logOR = res$se,
 z_value = res$zval,
 p_value = res$pval,
 Q = res$QE,
 Q_p = res$QEp,
 I2 = res$I2,
 tau2 = res$tau2,
 Pred_Lower = if(!is.null(res$pi.lb)) exp(res$pi.lb) else NA,
 Pred_Upper = if(!is.null(res$pi.ub)) exp(res$pi.ub) else NA
 )
}

results <- rbind(
 extract_ma_results(res_OR, "Socioeconomic/Educational Disadvantage Composite"),
 extract_ma_results(res_Counterpart, "Socioeconomic/Educational Disadvantage Composite (Counterpart)")
)

write_xlsx(results, "MA_Parental_Composite_Progression_07_11_2025.xlsx")


#Metaanalysis "Parental Income on Progression"

#Step1
subset_data <- data[
 data$predictor == "Socioeconomic/Educational Disadvantage Composite" &
 data$predictor_indicator == "Parental Income" &
 data$outcome == "Progression",
 ]
names(subset_data)

## [1] "Code" "key"
## [3] "author" "title"
## [5] "Field" "cohort"
## [7] "Date_range" "predictor"
## [9] "predictor_indicator" "outcome"
## [11] "outcome_indicator" "Effect_direction"
## [13] "Effect_Description" "Effect_type (Cohen's d, OR, etc)"
## [15] "Effect_size" "Lower_CI"
## [17] "Upper_CI" "n_applicant"
## [19] "n_selected" "n_enrollee"
## [21] "n_progressed" "logOR"
## [23] "SE_logOR" "Counterpart_OR"
## [25] "Counterpart_Lower_CI" "Counterpart_Upper_CI"
## [27] "logCounterpart_OR" "SE_logCounterpart_OR"

##Step2: "Being from a low income family"
library(metafor)
res_OR <- rma(
 yi = subset_data$logOR,
 sei = subset_data$SE_logOR,
 method = "REML",
 slab = subset_data$author
)
summary(res_OR)

##
## Random-Effects Model (k = 3; tau^2 estimator: REML)
##
## logLik deviance AIC BIC AICc
## 2.1787 -4.3574 -0.3574 -2.9711 11.6426
##
## tau^2 (estimated amount of total heterogeneity): 0 (SE = 0.0049)
## tau (square root of estimated tau^2 value): 0
## I^2 (total heterogeneity / total variability): 0.00%
## H^2 (total variability / sampling variability): 1.00
##
## Test for Heterogeneity:
## Q(df = 2) = 0.0698, p-val = 0.9657
##
## Model Results:
##
## estimate se zval pval ci.lb ci.ub
## -0.5989 0.0384 -15.5934 <.0001 -0.6742 -0.5236 ***
##
## ---
## Signif. codes: 0 '***' 0.001 '**' 0.01 '*' 0.05 '.' 0.1 ' ' 1

###Step3: "Not being from a low income family"
res_Counterpart <- rma(
 yi = subset_data$logCounterpart_OR,
 sei = subset_data$SE_logCounterpart_OR,
 method = "REML",
 slab = subset_data$author
)
summary(res_Counterpart)

##
## Random-Effects Model (k = 3; tau^2 estimator: REML)
##
## logLik deviance AIC BIC AICc
## 2.1787 -4.3574 -0.3574 -2.9711 11.6426
##
## tau^2 (estimated amount of total heterogeneity): 0 (SE = 0.0049)
## tau (square root of estimated tau^2 value): 0
## I^2 (total heterogeneity / total variability): 0.00%
## H^2 (total variability / sampling variability): 1.00
##
## Test for Heterogeneity:
## Q(df = 2) = 0.0698, p-val = 0.9657
##
## Model Results:
##
## estimate se zval pval ci.lb ci.ub
## 0.5989 0.0384 15.5934 <.0001 0.5236 0.6742 ***
##
## ---
## Signif. codes: 0 '***' 0.001 '**' 0.01 '*' 0.05 '.' 0.1 ' ' 1

###Step4:
library(writexl)

# Helper function to extract meta-analysis summary
extract_ma_results <- function(res, label) {
 data.frame(
 Effect = label,
 Model = res$method, # e.g., REML
 Studies = res$k,
 Estimate = exp(res$b),
 Lower_CI = exp(res$ci.lb),
 Upper_CI = exp(res$ci.ub),
 logOR = res$b,
 SE_logOR = res$se,
 z_value = res$zval,
 p_value = res$pval,
 Q = res$QE,
 Q_p = res$QEp,
 I2 = res$I2,
 tau2 = res$tau2,
 Pred_Lower = if(!is.null(res$pi.lb)) exp(res$pi.lb) else NA,
 Pred_Upper = if(!is.null(res$pi.ub)) exp(res$pi.ub) else NA
 )
}

results <- rbind(
 extract_ma_results(res_OR, "Low Parental Income"),
 extract_ma_results(res_Counterpart, "Low Parental Income (Counterpart)")
)

write_xlsx(results, "MA_Parental_Income_Progression_07_11_2025.xlsx")


#Metaanalysis "Parental Education (being first gen) on Progression"

#Step1
subset_data <- data[
 data$predictor == "Socioeconomic/Educational Disadvantage Composite" &
 data$predictor_indicator == "Parental Education (First generation university applicant)" &
 data$outcome == "Progression",
 ]
names(subset_data)

## [1] "Code" "key"
## [3] "author" "title"
## [5] "Field" "cohort"
## [7] "Date_range" "predictor"
## [9] "predictor_indicator" "outcome"
## [11] "outcome_indicator" "Effect_direction"
## [13] "Effect_Description" "Effect_type (Cohen's d, OR, etc)"
## [15] "Effect_size" "Lower_CI"
## [17] "Upper_CI" "n_applicant"
## [19] "n_selected" "n_enrollee"
## [21] "n_progressed" "logOR"
## [23] "SE_logOR" "Counterpart_OR"
## [25] "Counterpart_Lower_CI" "Counterpart_Upper_CI"
## [27] "logCounterpart_OR" "SE_logCounterpart_OR"

##Step2: "First generation"
library(metafor)
res_OR <- rma(
 yi = subset_data$logOR,
 sei = subset_data$SE_logOR,
 method = "REML",
 slab = subset_data$author
)
summary(res_OR)

##
## Random-Effects Model (k = 3; tau^2 estimator: REML)
##
## logLik deviance AIC BIC AICc
## 1.7810 -3.5620 0.4380 -2.1757 12.4380
##
## tau^2 (estimated amount of total heterogeneity): 0.0017 (SE = 0.0069)
## tau (square root of estimated tau^2 value): 0.0406
## I^2 (total heterogeneity / total variability): 21.41%
## H^2 (total variability / sampling variability): 1.27
##
## Test for Heterogeneity:
## Q(df = 2) = 1.6656, p-val = 0.4348
##
## Model Results:
##
## estimate se zval pval ci.lb ci.ub
## -0.5523 0.0482 -11.4624 <.0001 -0.6467 -0.4579 ***
##
## ---
## Signif. codes: 0 '***' 0.001 '**' 0.01 '*' 0.05 '.' 0.1 ' ' 1

###Step3: "Not first generation"
res_Counterpart <- rma(
 yi = subset_data$logCounterpart_OR,
 sei = subset_data$SE_logCounterpart_OR,
 method = "REML",
 slab = subset_data$author
)
summary(res_Counterpart)

##
## Random-Effects Model (k = 3; tau^2 estimator: REML)
##
## logLik deviance AIC BIC AICc
## 1.7810 -3.5620 0.4380 -2.1757 12.4380
##
## tau^2 (estimated amount of total heterogeneity): 0.0017 (SE = 0.0069)
## tau (square root of estimated tau^2 value): 0.0406
## I^2 (total heterogeneity / total variability): 21.41%
## H^2 (total variability / sampling variability): 1.27
##
## Test for Heterogeneity:
## Q(df = 2) = 1.6656, p-val = 0.4348
##
## Model Results:
##
## estimate se zval pval ci.lb ci.ub
## 0.5523 0.0482 11.4624 <.0001 0.4579 0.6467 ***
##
## ---
## Signif. codes: 0 '***' 0.001 '**' 0.01 '*' 0.05 '.' 0.1 ' ' 1

###Step4:
library(writexl)

# Helper function to extract meta-analysis summary
extract_ma_results <- function(res, label) {
 data.frame(
 Effect = label,
 Model = res$method, # e.g., REML
 Studies = res$k,
 Estimate = exp(res$b),
 Lower_CI = exp(res$ci.lb),
 Upper_CI = exp(res$ci.ub),
 logOR = res$b,
 SE_logOR = res$se,
 z_value = res$zval,
 p_value = res$pval,
 Q = res$QE,
 Q_p = res$QEp,
 I2 = res$I2,
 tau2 = res$tau2,
 Pred_Lower = if(!is.null(res$pi.lb)) exp(res$pi.lb) else NA,
 Pred_Upper = if(!is.null(res$pi.ub)) exp(res$pi.ub) else NA
 )
}

results <- rbind(
 extract_ma_results(res_OR, "First Generation"),
 extract_ma_results(res_Counterpart, "First Generation (Counterpart)")
)

write_xlsx(results, "MA_First_Gen_Progression_07_11_2025.xlsx")


#Metaanalysis "Geographic area on Progression"

#Step1
subset_data <- data[
 data$predictor == "Geographic area" &
 data$outcome == "Progression",
 ]
names(subset_data)

## [1] "Code" "key"
## [3] "author" "title"
## [5] "Field" "cohort"
## [7] "Date_range" "predictor"
## [9] "predictor_indicator" "outcome"
## [11] "outcome_indicator" "Effect_direction"
## [13] "Effect_Description" "Effect_type (Cohen's d, OR, etc)"
## [15] "Effect_size" "Lower_CI"
## [17] "Upper_CI" "n_applicant"
## [19] "n_selected" "n_enrollee"
## [21] "n_progressed" "logOR"
## [23] "SE_logOR" "Counterpart_OR"
## [25] "Counterpart_Lower_CI" "Counterpart_Upper_CI"
## [27] "logCounterpart_OR" "SE_logCounterpart_OR"

##Step2: "Being from an underesourced area"
library(metafor)
res_OR <- rma(
 yi = subset_data$logOR,
 sei = subset_data$SE_logOR,
 method = "REML",
 slab = subset_data$author
)
summary(res_OR)

##
## Random-Effects Model (k = 1; tau^2 estimator: REML)
##
## logLik deviance AIC BIC AICc
## -0.0000 0.0000 4.0000 -Inf 16.0000
##
## tau^2 (estimated amount of total heterogeneity): 0
## tau (square root of estimated tau^2 value): 0
## I^2 (total heterogeneity / total variability): 0.00%
## H^2 (total variability / sampling variability): 1.00
##
## Test for Heterogeneity:
## Q(df = 0) = 0.0000, p-val = 1.0000
##
## Model Results:
##
## estimate se zval pval ci.lb ci.ub
## -0.6931 0.0763 -9.0800 <.0001 -0.8428 -0.5435 ***
##
## ---
## Signif. codes: 0 '***' 0.001 '**' 0.01 '*' 0.05 '.' 0.1 ' ' 1

###Step3: "Not being from an underesourced area"
res_Counterpart <- rma(
 yi = subset_data$logCounterpart_OR,
 sei = subset_data$SE_logCounterpart_OR,
 method = "REML",
 slab = subset_data$author
)
summary(res_Counterpart)

##
## Random-Effects Model (k = 1; tau^2 estimator: REML)
##
## logLik deviance AIC BIC AICc
## -0.0000 0.0000 4.0000 -Inf 16.0000
##
## tau^2 (estimated amount of total heterogeneity): 0
## tau (square root of estimated tau^2 value): 0
## I^2 (total heterogeneity / total variability): 0.00%
## H^2 (total variability / sampling variability): 1.00
##
## Test for Heterogeneity:
## Q(df = 0) = 0.0000, p-val = 1.0000
##
## Model Results:
##
## estimate se zval pval ci.lb ci.ub
## 0.6931 0.0763 9.0800 <.0001 0.5435 0.8428 ***
##
## ---
## Signif. codes: 0 '***' 0.001 '**' 0.01 '*' 0.05 '.' 0.1 ' ' 1

###Step4:
library(writexl)

# Helper function to extract meta-analysis summary
extract_ma_results <- function(res, label) {
 data.frame(
 Effect = label,
 Model = res$method, # e.g., REML
 Studies = res$k,
 Estimate = exp(res$b),
 Lower_CI = exp(res$ci.lb),
 Upper_CI = exp(res$ci.ub),
 logOR = res$b,
 SE_logOR = res$se,
 z_value = res$zval,
 p_value = res$pval,
 Q = res$QE,
 Q_p = res$QEp,
 I2 = res$I2,
 tau2 = res$tau2,
 Pred_Lower = if(!is.null(res$pi.lb)) exp(res$pi.lb) else NA,
 Pred_Upper = if(!is.null(res$pi.ub)) exp(res$pi.ub) else NA
 )
}

results <- rbind(
 extract_ma_results(res_OR, "Deprived Area"),
 extract_ma_results(res_Counterpart, "Deprived Area (Counterpart)")
)

write_xlsx(results, "MA_Geographic_area_Progression_07_11_2025.xlsx")


#Metaanalysis "Geographic area (combined) on Selection"

#Step1
subset_data <- data[
 data$predictor == "Geographic area" &
 data$outcome == "Selection",
 ]
names(subset_data)

## [1] "Code" "key"
## [3] "author" "title"
## [5] "Field" "cohort"
## [7] "Date_range" "predictor"
## [9] "predictor_indicator" "outcome"
## [11] "outcome_indicator" "Effect_direction"
## [13] "Effect_Description" "Effect_type (Cohen's d, OR, etc)"
## [15] "Effect_size" "Lower_CI"
## [17] "Upper_CI" "n_applicant"
## [19] "n_selected" "n_enrollee"
## [21] "n_progressed" "logOR"
## [23] "SE_logOR" "Counterpart_OR"
## [25] "Counterpart_Lower_CI" "Counterpart_Upper_CI"
## [27] "logCounterpart_OR" "SE_logCounterpart_OR"

##Step2: "Being from a Deprived Area"
library(metafor)
res_OR <- rma(
 yi = subset_data$logOR,
 sei = subset_data$SE_logOR,
 method = "REML",
 slab = subset_data$author
)
summary(res_OR)

##
## Random-Effects Model (k = 6; tau^2 estimator: REML)
##
## logLik deviance AIC BIC AICc
## -2.5395 5.0791 9.0791 8.2979 15.0791
##
## tau^2 (estimated amount of total heterogeneity): 0.1427 (SE = 0.0953)
## tau (square root of estimated tau^2 value): 0.3777
## I^2 (total heterogeneity / total variability): 99.27%
## H^2 (total variability / sampling variability): 137.83
##
## Test for Heterogeneity:
## Q(df = 5) = 1067.9635, p-val < .0001
##
## Model Results:
##
## estimate se zval pval ci.lb ci.ub
## -0.3763 0.1587 -2.3719 0.0177 -0.6873 -0.0654 *
##
## ---
## Signif. codes: 0 '***' 0.001 '**' 0.01 '*' 0.05 '.' 0.1 ' ' 1

###Step3: "Not being from a Deprived Area"
res_Counterpart <- rma(
 yi = subset_data$logCounterpart_OR,
 sei = subset_data$SE_logCounterpart_OR,
 method = "REML",
 slab = subset_data$author
)
summary(res_Counterpart)

##
## Random-Effects Model (k = 6; tau^2 estimator: REML)
##
## logLik deviance AIC BIC AICc
## -2.5395 5.0791 9.0791 8.2979 15.0791
##
## tau^2 (estimated amount of total heterogeneity): 0.1427 (SE = 0.0953)
## tau (square root of estimated tau^2 value): 0.3777
## I^2 (total heterogeneity / total variability): 99.27%
## H^2 (total variability / sampling variability): 137.83
##
## Test for Heterogeneity:
## Q(df = 5) = 1067.9635, p-val < .0001
##
## Model Results:
##
## estimate se zval pval ci.lb ci.ub
## 0.3763 0.1587 2.3719 0.0177 0.0654 0.6873 *
##
## ---
## Signif. codes: 0 '***' 0.001 '**' 0.01 '*' 0.05 '.' 0.1 ' ' 1

###Step4:
library(writexl)

# Helper function to extract meta-analysis summary
extract_ma_results <- function(res, label) {
 data.frame(
 Effect = label,
 Model = res$method, # e.g., REML
 Studies = res$k,
 Estimate = exp(res$b),
 Lower_CI = exp(res$ci.lb),
 Upper_CI = exp(res$ci.ub),
 logOR = res$b,
 SE_logOR = res$se,
 z_value = res$zval,
 p_value = res$pval,
 Q = res$QE,
 Q_p = res$QEp,
 I2 = res$I2,
 tau2 = res$tau2,
 Pred_Lower = if(!is.null(res$pi.lb)) exp(res$pi.lb) else NA,
 Pred_Upper = if(!is.null(res$pi.ub)) exp(res$pi.ub) else NA
 )
}

results <- rbind(
 extract_ma_results(res_OR, "Deprived Area"),
 extract_ma_results(res_Counterpart, "Deprived Area (Counterpart)")
)

write_xlsx(results, "MA_Geographic_Area_Selection_07_11_2025.xlsx")

#Metaanalysis "Parental Socioeconomic/Educational Disadvantage Composite on Selection"

#Step1
subset_data <- data[
 data$predictor == "Socioeconomic/Educational Disadvantage Composite" &
 data$outcome == "Selection",
 ]
names(subset_data)

## [1] "Code" "key"
## [3] "author" "title"
## [5] "Field" "cohort"
## [7] "Date_range" "predictor"
## [9] "predictor_indicator" "outcome"
## [11] "outcome_indicator" "Effect_direction"
## [13] "Effect_Description" "Effect_type (Cohen's d, OR, etc)"
## [15] "Effect_size" "Lower_CI"
## [17] "Upper_CI" "n_applicant"
## [19] "n_selected" "n_enrollee"
## [21] "n_progressed" "logOR"
## [23] "SE_logOR" "Counterpart_OR"
## [25] "Counterpart_Lower_CI" "Counterpart_Upper_CI"
## [27] "logCounterpart_OR" "SE_logCounterpart_OR"

##Step2: "Being from a Low-SES Family (combined)"
library(metafor)
res_OR <- rma(
 yi = subset_data$logOR,
 sei = subset_data$SE_logOR,
 method = "REML",
 slab = subset_data$author
)
summary(res_OR)

##
## Random-Effects Model (k = 22; tau^2 estimator: REML)
##
## logLik deviance AIC BIC AICc
## 0.8443 -1.6887 2.3113 4.4004 2.9780
##
## tau^2 (estimated amount of total heterogeneity): 0.0285 (SE = 0.0109)
## tau (square root of estimated tau^2 value): 0.1689
## I^2 (total heterogeneity / total variability): 98.68%
## H^2 (total variability / sampling variability): 75.90
##
## Test for Heterogeneity:
## Q(df = 21) = 575.1837, p-val < .0001
##
## Model Results:
##
## estimate se zval pval ci.lb ci.ub
## -0.5122 0.0415 -12.3301 <.0001 -0.5936 -0.4308 ***
##
## ---
## Signif. codes: 0 '***' 0.001 '**' 0.01 '*' 0.05 '.' 0.1 ' ' 1

###Step3: "Not being from a Low-SES Family (combined)"
res_Counterpart <- rma(
 yi = subset_data$logCounterpart_OR,
 sei = subset_data$SE_logCounterpart_OR,
 method = "REML",
 slab = subset_data$author
)
summary(res_Counterpart)

##
## Random-Effects Model (k = 22; tau^2 estimator: REML)
##
## logLik deviance AIC BIC AICc
## 0.8443 -1.6887 2.3113 4.4004 2.9780
##
## tau^2 (estimated amount of total heterogeneity): 0.0285 (SE = 0.0109)
## tau (square root of estimated tau^2 value): 0.1689
## I^2 (total heterogeneity / total variability): 98.68%
## H^2 (total variability / sampling variability): 75.90
##
## Test for Heterogeneity:
## Q(df = 21) = 575.1837, p-val < .0001
##
## Model Results:
##
## estimate se zval pval ci.lb ci.ub
## 0.5122 0.0415 12.3301 <.0001 0.4308 0.5936 ***
##
## ---
## Signif. codes: 0 '***' 0.001 '**' 0.01 '*' 0.05 '.' 0.1 ' ' 1

###Step4:
library(writexl)

# Helper function to extract meta-analysis summary
extract_ma_results <- function(res, label) {
 data.frame(
 Effect = label,
 Model = res$method, # e.g., REML
 Studies = res$k,
 Estimate = exp(res$b),
 Lower_CI = exp(res$ci.lb),
 Upper_CI = exp(res$ci.ub),
 logOR = res$b,
 SE_logOR = res$se,
 z_value = res$zval,
 p_value = res$pval,
 Q = res$QE,
 Q_p = res$QEp,
 I2 = res$I2,
 tau2 = res$tau2,
 Pred_Lower = if(!is.null(res$pi.lb)) exp(res$pi.lb) else NA,
 Pred_Upper = if(!is.null(res$pi.ub)) exp(res$pi.ub) else NA
 )
}

results <- rbind(
 extract_ma_results(res_OR, "Socioeconomic/Educational Disadvantage Composite"),
 extract_ma_results(res_Counterpart, "Socioeconomic/Educational Disadvantage Composite (Counterpart)")
)

write_xlsx(results, "MA_Parental_education_occupation_Combined_Selection_07_11_2025.xlsx")

# ------------------------------------------------------------------------

# Publication Bias Assessment: Funnel Plot and Egger's Test

# For: Socioeconomic/Educational Disadvantage Composite on Selection (k=22)

# ------------------------------------------------------------------------

library(metafor)

library(writexl)

# 1. Generate and Save the Funnel Plot as a PNG

png("Funnel_Plot_Composite_Selection.png", width=1800, height=1400, res=300)

funnel(res_OR,

main="Household Economic and Educational Disadvantage",

xlab="Log Odds Ratio",

ylab="Standard Error")

dev.off()

# 2. Perform Egger's Test (Regression test for funnel plot asymmetry)

egger_test <- regtest(res_OR, model="rma", predictor="sei")

# 3. Extract the results into a data frame

egger_results <- data.frame(

Analysis = "Composite Disadvantage on Selection (k=22)",

Test = "Egger's Test for Funnel Plot Asymmetry",

z_value = egger_test$zval,

p_value = egger_test$pval

)

# 4. Save the Egger's test results to an Excel file

write_xlsx(egger_results, "Eggers_Test_Composite_Selection.xlsx")

print(egger_results)

#Metaanalysis "Parental Income (low income) on Selection"

#Step1
subset_data <- data[
 data$predictor == "Socioeconomic/Educational Disadvantage Composite" &
 data$predictor_indicator == "Parental Income" &
 data$outcome == "Selection",
 ]
names(subset_data)

## [1] "Code" "key"
## [3] "author" "title"
## [5] "Field" "cohort"
## [7] "Date_range" "predictor"
## [9] "predictor_indicator" "outcome"
## [11] "outcome_indicator" "Effect_direction"
## [13] "Effect_Description" "Effect_type (Cohen's d, OR, etc)"
## [15] "Effect_size" "Lower_CI"
## [17] "Upper_CI" "n_applicant"
## [19] "n_selected" "n_enrollee"
## [21] "n_progressed" "logOR"
## [23] "SE_logOR" "Counterpart_OR"
## [25] "Counterpart_Lower_CI" "Counterpart_Upper_CI"
## [27] "logCounterpart_OR" "SE_logCounterpart_OR"

##Step2: "Being from a low-income family"
library(metafor)
res_OR <- rma(
 yi = subset_data$logOR,
 sei = subset_data$SE_logOR,
 method = "REML",
 slab = subset_data$author
)
summary(res_OR)

##
## Random-Effects Model (k = 3; tau^2 estimator: REML)
##
## logLik deviance AIC BIC AICc
## -0.0558 0.1115 4.1115 1.4978 16.1115
##
## tau^2 (estimated amount of total heterogeneity): 0.0611 (SE = 0.0616)
## tau (square root of estimated tau^2 value): 0.2472
## I^2 (total heterogeneity / total variability): 99.72%
## H^2 (total variability / sampling variability): 352.88
##
## Test for Heterogeneity:
## Q(df = 2) = 234.8634, p-val < .0001
##
## Model Results:
##
## estimate se zval pval ci.lb ci.ub
## -0.5677 0.1433 -3.9624 <.0001 -0.8485 -0.2869 ***
##
## ---
## Signif. codes: 0 '***' 0.001 '**' 0.01 '*' 0.05 '.' 0.1 ' ' 1

###Step3: "Not being from a low-income family"
res_Counterpart <- rma(
 yi = subset_data$logCounterpart_OR,
 sei = subset_data$SE_logCounterpart_OR,
 method = "REML",
 slab = subset_data$author
)
summary(res_Counterpart)

##
## Random-Effects Model (k = 3; tau^2 estimator: REML)
##
## logLik deviance AIC BIC AICc
## -0.0558 0.1115 4.1115 1.4978 16.1115
##
## tau^2 (estimated amount of total heterogeneity): 0.0611 (SE = 0.0616)
## tau (square root of estimated tau^2 value): 0.2472
## I^2 (total heterogeneity / total variability): 99.72%
## H^2 (total variability / sampling variability): 352.88
##
## Test for Heterogeneity:
## Q(df = 2) = 234.8634, p-val < .0001
##
## Model Results:
##
## estimate se zval pval ci.lb ci.ub
## 0.5677 0.1433 3.9624 <.0001 0.2869 0.8485 ***
##
## ---
## Signif. codes: 0 '***' 0.001 '**' 0.01 '*' 0.05 '.' 0.1 ' ' 1

###Step4:
library(writexl)

# Helper function to extract meta-analysis summary
extract_ma_results <- function(res, label) {
 data.frame(
 Effect = label,
 Model = res$method, # e.g., REML
 Studies = res$k,
 Estimate = exp(res$b),
 Lower_CI = exp(res$ci.lb),
 Upper_CI = exp(res$ci.ub),
 logOR = res$b,
 SE_logOR = res$se,
 z_value = res$zval,
 p_value = res$pval,
 Q = res$QE,
 Q_p = res$QEp,
 I2 = res$I2,
 tau2 = res$tau2,
 Pred_Lower = if(!is.null(res$pi.lb)) exp(res$pi.lb) else NA,
 Pred_Upper = if(!is.null(res$pi.ub)) exp(res$pi.ub) else NA
 )
}

results <- rbind(
 extract_ma_results(res_OR, "Low Parental Income"),
 extract_ma_results(res_Counterpart, "Low Parental Income (Counterpart)")
)

write_xlsx(results, "MA_Parental_Income_Selection_07_11_2025.xlsx")


#Metaanalysis "Parental Occupation on Selection"

#Step1
subset_data <- data[
 data$predictor == "Socioeconomic/Educational Disadvantage Composite" &
 data$predictor_indicator == "Parental Occupation" &
 data$outcome == "Selection",
 ]
names(subset_data)

## [1] "Code" "key"
## [3] "author" "title"
## [5] "Field" "cohort"
## [7] "Date_range" "predictor"
## [9] "predictor_indicator" "outcome"
## [11] "outcome_indicator" "Effect_direction"
## [13] "Effect_Description" "Effect_type (Cohen's d, OR, etc)"
## [15] "Effect_size" "Lower_CI"
## [17] "Upper_CI" "n_applicant"
## [19] "n_selected" "n_enrollee"
## [21] "n_progressed" "logOR"
## [23] "SE_logOR" "Counterpart_OR"
## [25] "Counterpart_Lower_CI" "Counterpart_Upper_CI"
## [27] "logCounterpart_OR" "SE_logCounterpart_OR"

##Step2: "Having Socioeconomically Disadvantaged Parental Occupation"
library(metafor)
res_OR <- rma(
 yi = subset_data$logOR,
 sei = subset_data$SE_logOR,
 method = "REML",
 slab = subset_data$author
)
summary(res_OR)

##
## Random-Effects Model (k = 9; tau^2 estimator: REML)
##
## logLik deviance AIC BIC AICc
## 3.2220 -6.4439 -2.4439 -2.2850 -0.0439
##
## tau^2 (estimated amount of total heterogeneity): 0.0054 (SE = 0.0046)
## tau (square root of estimated tau^2 value): 0.0736
## I^2 (total heterogeneity / total variability): 75.01%
## H^2 (total variability / sampling variability): 4.00
##
## Test for Heterogeneity:
## Q(df = 8) = 25.7344, p-val = 0.0012
##
## Model Results:
##
## estimate se zval pval ci.lb ci.ub
## -0.6163 0.0346 -17.8084 <.0001 -0.6842 -0.5485 ***
##
## ---
## Signif. codes: 0 '***' 0.001 '**' 0.01 '*' 0.05 '.' 0.1 ' ' 1

###Step3: "Not having Socioeconomically Disadvantaged Parental Occupation"
res_Counterpart <- rma(
 yi = subset_data$logCounterpart_OR,
 sei = subset_data$SE_logCounterpart_OR,
 method = "REML",
 slab = subset_data$author
)
summary(res_Counterpart)

##
## Random-Effects Model (k = 9; tau^2 estimator: REML)
##
## logLik deviance AIC BIC AICc
## 3.2220 -6.4439 -2.4439 -2.2850 -0.0439
##
## tau^2 (estimated amount of total heterogeneity): 0.0054 (SE = 0.0046)
## tau (square root of estimated tau^2 value): 0.0736
## I^2 (total heterogeneity / total variability): 75.01%
## H^2 (total variability / sampling variability): 4.00
##
## Test for Heterogeneity:
## Q(df = 8) = 25.7344, p-val = 0.0012
##
## Model Results:
##
## estimate se zval pval ci.lb ci.ub
## 0.6163 0.0346 17.8084 <.0001 0.5485 0.6842 ***
##
## ---
## Signif. codes: 0 '***' 0.001 '**' 0.01 '*' 0.05 '.' 0.1 ' ' 1

###Step4:
library(writexl)

# Helper function to extract meta-analysis summary
extract_ma_results <- function(res, label) {
 data.frame(
 Effect = label,
 Model = res$method, # e.g., REML
 Studies = res$k,
 Estimate = exp(res$b),
 Lower_CI = exp(res$ci.lb),
 Upper_CI = exp(res$ci.ub),
 logOR = res$b,
 SE_logOR = res$se,
 z_value = res$zval,
 p_value = res$pval,
 Q = res$QE,
 Q_p = res$QEp,
 I2 = res$I2,
 tau2 = res$tau2,
 Pred_Lower = if(!is.null(res$pi.lb)) exp(res$pi.lb) else NA,
 Pred_Upper = if(!is.null(res$pi.ub)) exp(res$pi.ub) else NA
 )
}

results <- rbind(
 extract_ma_results(res_OR, "Socioeconomically Disadvantaged Parental Occupation"),
 extract_ma_results(res_Counterpart, "Socioeconomically Disadvantaged Parental Occupation (Counterpart)")
)

write_xlsx(results, "MA_Parental_Occupation_Selection_07_11_2025.xlsx")


#Metaanalysis "Parental Education on Selection"

#Step1
subset_data <- data[
 data$predictor == "Socioeconomic/Educational Disadvantage Composite" &
 data$predictor_indicator == "Parental Education (First generation university applicant)" &
 data$outcome == "Selection",
 ]
names(subset_data)

## [1] "Code" "key"
## [3] "author" "title"
## [5] "Field" "cohort"
## [7] "Date_range" "predictor"
## [9] "predictor_indicator" "outcome"
## [11] "outcome_indicator" "Effect_direction"
## [13] "Effect_Description" "Effect_type (Cohen's d, OR, etc)"
## [15] "Effect_size" "Lower_CI"
## [17] "Upper_CI" "n_applicant"
## [19] "n_selected" "n_enrollee"
## [21] "n_progressed" "logOR"
## [23] "SE_logOR" "Counterpart_OR"
## [25] "Counterpart_Lower_CI" "Counterpart_Upper_CI"
## [27] "logCounterpart_OR" "SE_logCounterpart_OR"

##Step2: "Being First Gen"
library(metafor)
res_OR <- rma(
 yi = subset_data$logOR,
 sei = subset_data$SE_logOR,
 method = "REML",
 slab = subset_data$author
)
summary(res_OR)

##
## Random-Effects Model (k = 4; tau^2 estimator: REML)
##
## logLik deviance AIC BIC AICc
## 1.9853 -3.9705 0.0295 -1.7733 12.0295
##
## tau^2 (estimated amount of total heterogeneity): 0.0090 (SE = 0.0097)
## tau (square root of estimated tau^2 value): 0.0949
## I^2 (total heterogeneity / total variability): 95.37%
## H^2 (total variability / sampling variability): 21.59
##
## Test for Heterogeneity:
## Q(df = 3) = 80.8707, p-val < .0001
##
## Model Results:
##
## estimate se zval pval ci.lb ci.ub
## -0.4747 0.0553 -8.5786 <.0001 -0.5832 -0.3663 ***
##
## ---
## Signif. codes: 0 '***' 0.001 '**' 0.01 '*' 0.05 '.' 0.1 ' ' 1

###Step3: "Not being first gen"
res_Counterpart <- rma(
 yi = subset_data$logCounterpart_OR,
 sei = subset_data$SE_logCounterpart_OR,
 method = "REML",
 slab = subset_data$author
)
summary(res_Counterpart)

##
## Random-Effects Model (k = 4; tau^2 estimator: REML)
##
## logLik deviance AIC BIC AICc
## 1.9853 -3.9705 0.0295 -1.7733 12.0295
##
## tau^2 (estimated amount of total heterogeneity): 0.0090 (SE = 0.0097)
## tau (square root of estimated tau^2 value): 0.0949
## I^2 (total heterogeneity / total variability): 95.37%
## H^2 (total variability / sampling variability): 21.59
##
## Test for Heterogeneity:
## Q(df = 3) = 80.8707, p-val < .0001
##
## Model Results:
##
## estimate se zval pval ci.lb ci.ub
## 0.4747 0.0553 8.5786 <.0001 0.3663 0.5832 ***
##
## ---
## Signif. codes: 0 '***' 0.001 '**' 0.01 '*' 0.05 '.' 0.1 ' ' 1

###Step4:
library(writexl)

# Helper function to extract meta-analysis summary
extract_ma_results <- function(res, label) {
 data.frame(
 Effect = label,
 Model = res$method, # e.g., REML
 Studies = res$k,
 Estimate = exp(res$b),
 Lower_CI = exp(res$ci.lb),
 Upper_CI = exp(res$ci.ub),
 logOR = res$b,
 SE_logOR = res$se,
 z_value = res$zval,
 p_value = res$pval,
 Q = res$QE,
 Q_p = res$QEp,
 I2 = res$I2,
 tau2 = res$tau2,
 Pred_Lower = if(!is.null(res$pi.lb)) exp(res$pi.lb) else NA,
 Pred_Upper = if(!is.null(res$pi.ub)) exp(res$pi.ub) else NA
 )
}

results <- rbind(
 extract_ma_results(res_OR, "First Generation"),
 extract_ma_results(res_Counterpart, "First Generation (Counterpart)")
)

write_xlsx(results, "MA_Parental_Education_Selection_07_11_2025.xlsx")


#Visualisation (Bar charts)

library(readxl)
library(ggplot2)

## Warning: package 'ggplot2' was built under R version 4.5.1

library(dplyr)

## Warning: package 'dplyr' was built under R version 4.5.1

##
## Attaching package: 'dplyr'

## The following objects are masked from 'package:stats':
##
## filter, lag

## The following objects are masked from 'package:base':
##
## intersect, setdiff, setequal, union

#Step 1: List of files for Progression and Selection
progression_files <- c(
 "MA_Debt_Progression_07_11_2025.xlsx",
 "MA_Parental_Composite_Progression_07_11_2025.xlsx",
 "MA_Parental_Income_Progression_07_11_2025.xlsx",
 "MA_Geographic_area_Progression_07_11_2025.xlsx",
 "MA_First_Gen_Progression_07_11_2025.xlsx"
)

progression_labels <- c(
 "Debt",
 "Socioeconomic/Educational Disadvantage Composite",
 "Low Parental Income",
 "Deprived Area",
 "First Generation"
)

selection_files <- c(
 "MA_Parental_Income_Selection_07_11_2025.xlsx",
 "MA_Geographic_Area_Selection_07_11_2025.xlsx",
 "MA_Parental_education_occupation_Combined_Selection_07_11_2025.xlsx",
 "MA_Parental_Occupation_Selection_07_11_2025.xlsx",
 "MA_Parental_Education_Selection_07_11_2025.xlsx"
)

selection_labels <- c(
 "Low Parental Income",
 "Deprived Area",
 "Socioeconomic/Educational Disadvantage Composite",
 "Socioeconomically Disadvantaged Parental Occupation",
 "First Generation"
)

#Step 2: Data Extraction function (with typo-tolerant matching)
extract_ma <- function(file, effect_label) {
 res <- read_excel(file)

 idx_exact <- which(tolower(trimws(res$Effect)) == tolower(trimws(effect_label)))

 if (length(idx_exact) == 1) {
 found <- res[idx_exact, ]
 } else {
 idx_approx <- agrep(effect_label, res$Effect, ignore.case = TRUE, max.distance = 0.15)
 if (length(idx_approx) > 0) {
 warning(paste("Effect label", effect_label, "was not exactly found in",
 file, ". Using nearest match:", res$Effect[idx_approx[1]]))
 found <- res[idx_approx[1], ]
 } else {
 warning(paste("Effect-", effect_label, "not found in", file))
 return(data.frame(
 Effect = effect_label,
 OR = NA,
 Lower_CI = NA,
 Upper_CI = NA,
 Studies = NA
 ))
 }
 }

 if (found$Studies[1] == 1) {
 warning(paste("Effect-", found$Effect, "in", file,
 "has only one study and will be excluded from plot."))
 return(data.frame(
 Effect = effect_label,
 OR = NA,
 Lower_CI = NA,
 Upper_CI = NA,
 Studies = found$Studies[1]
 ))
 }

 data.frame(
 Effect = effect_label,
 OR = found$Estimate[1],
 Lower_CI = found$Lower_CI[1],
 Upper_CI = found$Upper_CI[1],
 Studies = found$Studies[1]
 )
}


#Step 3: Read and Combine - exclude k=1 from plot

progression_data <- data.frame()
for (i in seq_along(progression_files)) {
 progression_data <- rbind(
 progression_data,
 extract_ma(progression_files[i], progression_labels[i])
 )
}

## Warning in extract_ma(progression_files[i], progression_labels[i]): Effect-
## Deprived Area in MA_Geographic_area_Progression_07_11_2025.xlsx has only one
## study and will be excluded from plot.

progression_data <- progression_data %>%
 filter(!is.na(OR)) %>%
 # Relabel effect names
 mutate(
 Effect = recode(
 Effect,
 "Debt" = "Premedical Debt",
 "Deprived Area" = "Deprived Geographic Area",
 "Socioeconomic/Educational Disadvantage Composite" = "Household Economic and Educational Disadvantage",
 "First Generation" = "First-Generation Status"
 ),
 EffectLabel = paste0(Effect, " (k=", Studies, ")")
 )
# Force the desired order in the bar chart for progression outcome
desired_order <- c("Premedical Debt",
 "Household Economic and Educational Disadvantage",
 "Low Parental Income",
 "Socioeconomically Disadvantaged Parental Occupation",
 "First-Generation Status")

progression_data$Effect <- factor(progression_data$Effect, levels = desired_order)
progression_data <- progression_data %>%
 arrange(match(Effect, desired_order)) %>%
 mutate(EffectLabel = paste0(Effect, " (k=", Studies, ")"))
progression_data$EffectLabel <- factor(
 progression_data$EffectLabel,
 levels = paste0(desired_order, " (k=", progression_data$Studies[match(desired_order, progression_data$Effect)], ")")
)

selection_data <- data.frame()
for (i in seq_along(selection_files)) {
 selection_data <- rbind(
 selection_data,
 extract_ma(selection_files[i], selection_labels[i])
 )
}

selection_data <- selection_data %>%
 mutate(
 Effect = recode(
 Effect,
 "Debt" = "Premedical Debt",
 "Deprived Area" = "Deprived Geographic Area",
 "Socioeconomic/Educational Disadvantage Composite" = "Household Economic and Educational Disadvantage",
 "First Generation" = "First-Generation Status"
 )
 )

# Force the desired order in the bar chart for selection outcome
desired_order <- c("Deprived Geographic Area",
 "Household Economic and Educational Disadvantage",
 "Low Parental Income",
 "Socioeconomically Disadvantaged Parental Occupation",
 "First-Generation Status")

selection_data$Effect <- factor(selection_data$Effect, levels = desired_order)
selection_data <- selection_data %>%
 arrange(match(Effect, desired_order)) %>%
 mutate(EffectLabel = paste0(Effect, " (k=", Studies, ")"))
selection_data$EffectLabel <- factor(
 selection_data$EffectLabel,
 levels = paste0(desired_order, " (k=", selection_data$Studies[match(desired_order, selection_data$Effect)], ")")
)

# Step 4: Plotting (study count now in x-axis label, not atop bar)

progression_plot <- ggplot(progression_data, aes(x = EffectLabel, y = OR, fill = EffectLabel)) +
 geom_bar(stat = "identity", width = 0.8) +
 geom_errorbar(aes(ymin = Lower_CI, ymax = Upper_CI), width = 0.3) +
 geom_text(aes(label = round(OR, 2)), vjust = -0.85, size = 4, hjust = -0.7) +
 ylab("Meta-analytic OR (95% CI)") +
 xlab("Disadvantaged Group (k=number of effect sizes)") +
 ggtitle("Meta-Analytic Odds Ratios for Progression Outcome") +
 theme_minimal() +
 theme(
 legend.position = "none",
 axis.text.x = element_text(angle=30, hjust=1),
 plot.margin = unit(c(1, 1, 1, 2), "cm") # top, right, bottom, left
 )

ggsave("Meta_Analytic_OR_Progression.png", progression_plot, width = 8, height = 6, dpi = 300)

selection_plot <- ggplot(selection_data, aes(x = EffectLabel, y = OR, fill = EffectLabel)) +
 geom_bar(stat = "identity", width = 0.8) +
 geom_errorbar(aes(ymin = Lower_CI, ymax = Upper_CI), width = 0.3) +
 geom_text(aes(label = round(OR, 2)), vjust = -0.85, size = 4, hjust = -0.7) +
 ylab("Meta-analytic OR (95% CI)") +
 xlab("Disadvantaged Group (k=number of effect sizes)") +
 ggtitle("Meta-Analytic Odds Ratios for Selection Outcome") +
 theme_minimal() +
 theme(
 legend.position = "none",
 axis.text.x = element_text(angle=30, hjust=1),
 plot.margin = unit(c(1, 1, 1, 2), "cm") # top, right, bottom, left
 )

ggsave("Meta_Analytic_OR_Selection.png", selection_plot, width = 8, height = 6, dpi = 300)


#Visualisation for heatmaps:


# Load required libraries
if (!requireNamespace("dplyr", quietly = TRUE)) install.packages("dplyr")
if (!requireNamespace("tidyr", quietly = TRUE)) install.packages("tidyr")
if (!requireNamespace("ggplot2", quietly = TRUE)) install.packages("ggplot2")
if (!requireNamespace("viridis", quietly = TRUE)) install.packages("viridis")

library(dplyr)
library(tidyr)

## Warning: package 'tidyr' was built under R version 4.5.1

##
## Attaching package: 'tidyr'

## The following objects are masked from 'package:Matrix':
##
## expand, pack, unpack

library(ggplot2)
library(viridis)

## Warning: package 'viridis' was built under R version 4.5.2

## Loading required package: viridisLite

## Warning: package 'viridisLite' was built under R version 4.5.1

# Step 1: List of files for Progression and Selection
progression_files <- c(
 "MA_Debt_Progression_07_11_2025.xlsx",
 "MA_Parental_Composite_Progression_07_11_2025.xlsx",
 "MA_Parental_Income_Progression_07_11_2025.xlsx",
 "MA_Geographic_area_Progression_07_11_2025.xlsx",
 "MA_First_Gen_Progression_07_11_2025.xlsx"
)

progression_labels <- c(
 "Debt",
 "Socioeconomic/Educational Disadvantage Composite",
 "Low Parental Income",
 "Deprived Area",
 "First Generation"
)

selection_files <- c(
 "MA_Parental_Income_Selection_07_11_2025.xlsx",
 "MA_Geographic_Area_Selection_07_11_2025.xlsx",
 "MA_Parental_education_occupation_Combined_Selection_07_11_2025.xlsx",
 "MA_Parental_Occupation_Selection_07_11_2025.xlsx",
 "MA_Parental_Education_Selection_07_11_2025.xlsx"
)

selection_labels <- c(
 "Low Parental Income",
 "Deprived Area",
 "Socioeconomic/Educational Disadvantage Composite",
 "Socioeconomically Disadvantaged Parental Occupation",
 "First Generation"
)

# Step 2: Extraction Function

extract_heatmap <- function(file, effect_label, counterpart = FALSE) {
 res <- readxl::read_excel(file)
 exact_idx <- which(tolower(trimws(res$Effect)) == tolower(trimws(effect_label)))
 if (length(exact_idx) == 1) {
 found <- res[exact_idx, ]
 } else {
 idx_approx <- agrep(effect_label, res$Effect, ignore.case = TRUE, max.distance = 0.15)
 if (length(idx_approx) > 0) {
 found <- res[idx_approx[1], ]
 } else {
 warning(paste(effect_label, "not found in", file))
 return(data.frame(Predictor=effect_label, Outcome=NA, OR=NA, Studies=NA, EffectType=ifelse(counterpart,"Counterpart","Disadvantaged")))
 }
 }
 if (found$Studies[1] == 1) {
 return(data.frame(Predictor=effect_label, Outcome=NA, OR=NA, Studies=1, EffectType=ifelse(counterpart,"Counterpart","Disadvantaged")))
 }
 if (counterpart) {
 or <- NA
 if(any(grepl("Counterpart", res$Effect, ignore.case = TRUE))){
 idx_counter <- grep("Counterpart", res$Effect, ignore.case = TRUE)
 or <- res$Estimate[idx_counter[1]]
 studies <- res$Studies[idx_counter[1]]
 return(data.frame(Predictor=effect_label, Outcome=NA, OR=or, Studies=studies, EffectType="Counterpart"))
 }
 return(data.frame(Predictor=effect_label, Outcome=NA, OR=NA, Studies=found$Studies[1], EffectType="Counterpart"))
 } else {
 return(data.frame(Predictor=effect_label, Outcome=NA, OR=found$Estimate[1], Studies=found$Studies[1], EffectType="Disadvantaged"))
 }
}

# Step 3: Build progression and selection heatmap data

progression_heatmap <- data.frame()
for (i in seq_along(progression_files)) {
 progression_heatmap <- rbind(
 progression_heatmap,
 extract_heatmap(progression_files[i], progression_labels[i], counterpart=FALSE) %>% dplyr::mutate(Outcome="Progression")
 )
 progression_heatmap <- rbind(
 progression_heatmap,
 extract_heatmap(progression_files[i], progression_labels[i], counterpart=TRUE) %>% dplyr::mutate(Outcome="Progression")
 )
}

selection_heatmap <- data.frame()
for (i in seq_along(selection_files)) {
 selection_heatmap <- rbind(
 selection_heatmap,
 extract_heatmap(selection_files[i], selection_labels[i], counterpart=FALSE) %>% dplyr::mutate(Outcome="Selection")
 )
 selection_heatmap <- rbind(
 selection_heatmap,
 extract_heatmap(selection_files[i], selection_labels[i], counterpart=TRUE) %>% dplyr::mutate(Outcome="Selection")
 )
}

# Combine all into one data frame and filter out NAs
hm_all <- rbind(progression_heatmap, selection_heatmap)
hm_all <- hm_all[!is.na(hm_all$OR), ]

# Step 4: Reshape for plotting

hm_long <- hm_all %>%
 dplyr::filter(EffectType == "Disadvantaged") %>%
 dplyr::select(Predictor, Outcome, OR, Studies)

#Relabelling
hm_long <- hm_long %>%
 mutate(Predictor = recode(
 Predictor,
 "Debt" = "Premedical Debt",
 "Deprived Area" = "Deprived Geographic Area",
 "Socioeconomic/Educational Disadvantage Composite" = "Household Economic and Educational Disadvantage",
 "First Generation" = "First-Generation Status"
 ))
desired_order <- c(
 "Premedical Debt",
 "Deprived Geographic Area",
 "Household Economic and Educational Disadvantage",
 "Low Parental Income",
 "Socioeconomically Disadvantaged Parental Occupation",
 "First-Generation Status"
)
hm_long <- hm_long %>%
 filter(Predictor %in% desired_order) %>%
 mutate(Predictor = factor(Predictor, levels = desired_order))
hm_long$Outcome <- factor(hm_long$Outcome, levels = c("Selection", "Progression"))

# Step 5: Create a generic continuous gradient for OR values

# Calculate the min/max and breakpoints for your ORs
min_or <- min(hm_long$OR, na.rm = TRUE)
max_or <- max(hm_long$OR, na.rm = TRUE)

# Use actual unique OR values for breaks and palette mapping
breaks <- sort(unique(hm_long$OR))
n_breaks <- length(breaks)

# Use a high-contrast gradient palette spanning your data
colours <- colorRampPalette(c("lightcyan", "paleturquoise", "lightskyblue", "deepskyblue", "royalblue", "mediumslateblue", "steelblue", "blue"))(n_breaks)

heatmap_disadvantaged <- ggplot(hm_long, aes(x = Outcome, y = Predictor, fill = OR)) +
 geom_tile(color = "white") +
 geom_text(
 aes(label = ifelse(is.na(OR), "", paste0(round(OR,2), "\n(k=",Studies,")"))),
 size = 4, color = "black"
 ) +
 scale_fill_gradientn(
 colours = colours,
 values = scales::rescale(breaks, from = c(min_or, max_or)), # forces colour switches at every observed OR
 limits = c(min_or, max_or),
 name = "OR (Disadvantaged)"
 ) +
 theme_minimal() +
 ggtitle("Meta-analytic Odds Ratios (Disadvantaged)\nPredictors × Outcomes") +
 ylab("Predictor") + xlab("Outcome")

ggsave("Heatmap_Disadvantaged_OR_07_11_2025.png", heatmap_disadvantaged, width = 9, height = 6, dpi = 300)

**References**

1. Bagg W, Curtis E, Eggleton KS, Nixon G, Bristowe Z, Brunton P, Hendry C, Kool B, Scarf D, Shaw S, Tukuitonga C, Williman J, Wilson D, Crampton P. Socio-demographic profile of medical students in Aotearoa, New Zealand (2016-2020): a nationwide cross-sectional study. BMJ Open 2023;13:e073996. <https://doi.org/10.1136/bmjopen-2023-073996>

2. Griffin B, Hu W. The interaction of socio-economic status and gender in widening participation in medicine. Med Educ 2015;49:103-113. <https://doi.org/10.1111/medu.12480>

3. James D, Ferguson E, Powis D, Symonds I, Yates J. Graduate entry to medicine: widening academic and socio-demographic access. Med Educ 2008;42:294-300. <https://doi.org/10.1111/j.1365-2923.2008.03006.x>

4. Zhang D, Li G, Mu L, Thapa J, Li Y, Chen Z, Shi L, Su D, Son H, Pagan JA. Trends in medical school application and matriculation rates across the United States from 2001 to 2015: implications for health disparities. Acad Med 2021;96:885-893. <https://doi.org/10.1097/ACM.0000000000004033>

5. Pitre T, Thomas A, Evans K, Jones A, Mountjoy M, Costa AP. The influence of income on medical school admissions in Canada: a retrospective cohort study. BMC Med Educ 2020;20:209. <https://doi.org/10.1186/s12909-020-02126-0>

6. Steven K, Dowell J, Jackson C, Guthrie B. Fair access to medicine?: retrospective analysis of UK medical schools application data 2009-2012 using three measures of socioeconomic status. BMC Med Educ 2016;16:11. <https://doi.org/10.1186/s12909-016-0536-1>

7. Taylor CA, Green KE, Spruce A. Evaluation of the effect of socio-economic status on performance in a Multiple Mini Interview for admission to medical school. Med Teach 2015;37:59-63. <https://doi.org/10.3109/0142159X.2014.923562>

8. Emery JL, Bell JF, Vidal Rodeiro CL. The BioMedical Admissions Test for medical student selection: issues of fairness and bias. Med Teach 2011;33:62-71. <https://doi.org/10.3109/0142159X.2010.528811>

9. Laurence CO, Turnbull DA, Briggs NE, Robinson JS. Applicant characteristics and their influence on success: results from an analysis of applicants to the University of Adelaide Medical School, 2004-2007. Med J Aust 2010;192:212-216. <https://doi.org/10.5694/j.1326-5377.2010.tb03481.x>

10. Lumsden MA, Bore M, Millar K, Jack R, Powis D. Assessment of personal qualities in relation to admission to medical school. Med Educ 2005;39:258-265. <https://doi.org/10.1111/j.1365-2929.2005.02087.x>

11. Kumwenda B, Cleland J, Greatrix R, MacKenzie RK, Prescott G. Are efforts to attract graduate applicants to UK medical schools effective in increasing the participation of under-represented socioeconomic groups?: a national cohort study. BMJ Open 2018;8:e018946. <https://doi.org/10.1136/bmjopen-2017-018946>

12. Nguyen M, Desai MM, Fancher TL, Chaudhry SI, Mason HR, Boatright D. Temporal trends in childhood household income among applicants and matriculants to medical school and the likelihood of acceptance by income, 2014-2019. JAMA 2023;329:1882-1884. <https://doi.org/10.1001/jama.2023.5654>

13. Perez MA, Williams C, Henderson K, McGregor R, Vapiwala N, Shea JA, Dine CJ. Association of applicant demographic factors with medical school acceptance. BMC Med Educ 2023;23:960. <https://doi.org/10.1186/s12909-023-04897-8>

14. Williams C, Perez MA, Vapiwala N, Shea JA. The impact of socioeconomic factors on medical school acceptance rates. Acad Med 2021;96:S219-S220. <https://doi.org/10.1097/ACM.0000000000004281>

15. Williams DK, Christophers B, Keyes T, Kumar R, Granovetter MC, Adigun A, Olivera J, Pura-Bryant J, Smith C, Okafor C, Shibre M, Daye D, Akabas MH. Sociodemographic factors and research experience impact MD-PhD program acceptance. JCI Insight 2024;9:e176146. <https://doi.org/10.1172/jci.insight.176146>

16. Mathers J, Sitch A, Parry J. Population-based longitudinal analyses of offer likelihood in UK medical schools: 1996-2012. Med Educ 2016;50:612-623. <https://doi.org/10.1111/medu.12981>

17. Mathers J, Sitch A, Parry J. Longitudinal assessment of the impact of the use of the UK clinical aptitude test for medical student selection. Med Educ 2016;50:1033-1044. <https://doi.org/10.1111/medu.13082>

18. Tiffin PA, Dowell JS, McLachlan JC. Widening access to UK medical education for under-represented socioeconomic groups: modelling the impact of the UKCAT in the 2009 cohort. BMJ 2012;344:e1805. <https://doi.org/10.1136/bmj.e1805>

19. Stegers-Jager KM, Steyerberg EW, Lucieer SM, Themmen AP. Ethnic and social disparities in performance on medical school selection criteria. Med Educ 2015;49:124-133. <https://doi.org/10.1111/medu.12536>

20. Finger C, Solga H, Elbers B. Social inequality in admission chances for prestigious higher education programs in Germany: do application patterns matter? Eur Sociol Rev 2024;40:1013-1029. <https://doi.org/10.1093/esr/jcae024>

21. Burbage AK, Hewitt EY. Exploring institutional stratification: minority-serving institutional pathways to medical school acceptance in the United States. Med Educ 2025;59:640-651. <https://doi.org/10.1111/medu.15539>

22. Ballejos MP, Oglesbee S, Hettema J, Sapien R. An equivalence study of interview platform: does videoconference technology impact medical school acceptance rates of different groups? Adv Health Sci Educ Theory Pract 2018;23:601-610. <https://doi.org/10.1007/s10459-018-9817-2>

23. Harrison LE, Fletcher L, Dunleavy D, Price-Johnson T, Vashi Kundu R, Fogerty GT, Berardi-Demo L. Self-reported disadvantage in medical school admissions: a call to review, revise, and further advance holistic review. Acad Med 2023;98:1044-1052. <https://doi.org/10.1097/ACM.0000000000005272>

24. Jerant A, Fancher T, Fenton JJ, Fiscella K, Sousa F, Franks P, Henderson M. How medical school applicant race, ethnicity, and socioeconomic status relate to multiple mini-interview-based admissions outcomes: findings from one medical school. Acad Med 2015;90:1667-1674. <https://doi.org/10.1097/ACM.0000000000000766>

25. Kennedy M. Medical school admissions across socioeconomic groups: an analysis across race neutral and race sensitive admissions cycles [dissertation]. University of North Texas; 2010.

26. Grbic D, Jones DJ, Case ST. The role of socioeconomic status in medical school admissions: validation of a socioeconomic indicator for use in medical school admissions. Acad Med 2015;90:953-960. <https://doi.org/10.1097/ACM.0000000000000653>

27. Andriole DA, Jeffe DB. Prematriculation variables associated with suboptimal outcomes for the 1994-1999 cohort of US medical school matriculants. JAMA 2010;304:1212-1219. <https://doi.org/10.1001/jama.2010.1321>

28. Jeffe DB, Andriole DA, Wathington HD, Tai RH. Educational outcomes for students enrolled in MD-PhD programs at medical school matriculation, 1995-2000: a national cohort study. Acad Med 2014;89:84-93. <https://doi.org/10.1097/ACM.0000000000000071>

29. Kamran SC, Pompa IR, Nguyen HB, Cha J, Salinas KE, Niemierko A, Vapiwala N. First-generation and low-income students in the national medical student body. JAMA Netw Open 2025;8:e259769. <https://doi.org/10.1001/jamanetworkopen.2025.9769>

30. Frischenschlager O, Haidinger G, Mitterauer L. Factors associated with academic success at Vienna Medical School: prospective survey. Croat Med J 2005;46:58-65.

31. Nguyen M, Chaudhry SI, Desai MM, Chen C, Mason HR, McDade WA, Fancher TL, Boatright D. Association of sociodemographic characteristics with US medical student attrition. JAMA Intern Med 2022;182:917-924. <https://doi.org/10.1001/jamainternmed.2022.2194>

32. Malau-Aduli BS, O’Connor T, Ray RA, van der Kruk Y, Bellingan M, Teague PA. Risk factors associated with academic difficulty in an Australian regionally located medical school. BMC Med Educ 2017;17:266. <https://doi.org/10.1186/s12909-017-1095-9>

33. Hanson JT, Busche K, Elks ML, Jackson-Williams LE, Liotta RA, Miller C, Morris CA, Thiessen B, Yuan K. The validity of MCAT scores in predicting students’ performance and progress in medical school: results from a multisite study. Acad Med 2022;97:1374-1384. <https://doi.org/10.1097/ACM.0000000000004754>
