# Supplementary material for: Socioeconomic and economic factors affecting access and progression in medical schools: a systematic review and meta-analysis
Source: J Educ Eval Health Prof. 2026 Apr 16;23:6. doi: 10.3352/jeehp.2026.23.6 (PMC13181141; doi:10.3352/jeehp.2026.23.6)
Supplement: Supplementary file 5 — Supplement 3. Record retrieval report. [file jeehp-23-06-suppl3.docx]

**Supplement 3.** Record retrieval report

| Database | Original records | Omitted after title deduplication | Omitted after DOI deduplication | Total duplicates omitted | Retained |
| --- | --- | --- | --- | --- | --- |
| Ebsco | 196 | 98 | 36 | 134 | 62 |
| Embase | 1,288 | 342 | 259 | 601 | 687 |
| ERIC | 43 | 5 | 16 | 21 | 22 |
| ProQuest | 20 | 0 | 3 | 3 | 17 |
| PubMed | 1,520 | 26 | 419 | 445 | 1,075 |
| Scopus | 961 | 2 | 0 | 2 | 959 |
| Total | 4,028 | 473 | 733 | 1,206 | 2,822 |
