# Supplementary material for: Socioeconomic and economic factors affecting access and progression in medical schools: a systematic review and meta-analysis
Source: J Educ Eval Health Prof. 2026 Apr 16;23:6. doi: 10.3352/jeehp.2026.23.6 (PMC13181141; doi:10.3352/jeehp.2026.23.6)
Supplement: Supplementary file 6 — Supplement 4. Impact of economic factors on students’ selection into medicine and dentistry programs. [file jeehp-23-06-suppl4.docx]

**Supplement 4.** Impact of economic factors on students’ selection into medicine programs

| No. | Category of economic factor | Predictor | Category of outcome | Outcome | Effect direction | Field | Title | Summary | Country | Author |
| --- | --- | --- | --- | --- | --- | --- | --- | --- | --- | --- |
| 1 | Geographic area | Socioeconomic deprivation (by home residence) | Selection | Enrolment | Negative^b)^ | Medicine | Socio-demographic profile of medical students in Aotearoa, New Zealand (2016–2020): a nationwide cross-sectional study | Higher socioeconomic deprivation is associated with lower enrolment rates.  Students from rural areas have lower enrolment rates than those from urban areas, independent of socioeconomic deprivation. | New Zealand | Bagg et al. [1] (2023) |
| 2 | Geographic area | Socioeconomic deprivation (by school catchment) | Selection | Enrolment | Negative^b)^ | Medicine | Socio-demographic profile of medical students in Aotearoa, New Zealand (2016–2020): a nationwide cross-sectional study | Enrolment distribution is skewed towards students from socioeconomically advantaged schools. | New Zealand | Bagg et al. [1] (2023) |
| 3 | Geographic area | Socioeconomic deprivation (by home residence) | Selection | Enrolment | Negative^b)^ | Medicine | The interaction of socio-economic status and gender in widening participation in medicine | Socioeconomic deprivation has a negative effect on enrolment in medicine. | Australia | Griffin & Hu [2] (2014) |
| 4 | Geographic area | Socioeconomic deprivation (by school catchment) | Selection | Enrolment | Negative^b)^ | Medicine | The interaction of socio-economic status and gender in widening participation in medicine | Majority of the enrolees came from schools in the less disadvantaged schools. | Australia | Griffin & Hu [2] (2014) |
| 5 | Geographic area | Socioeconomic deprivation (by home residence) | Selection | Acceptance | Negative^b)^ | Medicine | Graduate entry to medicine: widening academic and socio-demographic access | Socioeconomic deprivation has a negative effect on enrolment in medicine, with entrants being more advantaged that rejected applicants. | The United Kingdom | James et al. [3] (2008) |
| 6 | Geographic area | Neighborhood income | Selection | Enrolment | Negative^b)^ | Medicine | Trends in medical school application and matriculation rates across the United States from 2001 to 2015: implications for health disparities | Higher county median family income was associated with higher enrolment (matriculation) rates. | The United States | Zhang et al. [4] (2021) |
| 7 | Geographic area | Neighborhood income | Selection | Offer of a place | Negative | Medicine | The influence of income on medical school admissions in Canada: a retrospective cohort study | Applicants from areas with lower neighborhood income had lower odds of receiving an offer compared to those from areas with higher neighborhood income. | Canada | Pitre et al. [5] (2020) |
| 8 | Geographic area | Socioeconomic deprivation (by home residence) | Selection | Acceptance | Negative | Medicine | Fair access to medicine? Retrospective analysis of UK medical schools application data 2009–2012 using three measures of socioeconomic status | Socioeconomic deprivation has a negative effect on the likelihood of receiving an offer of a place in medical school. | The United Kingdom | Steven et al. [6] (2016) |
| 9 | Geographic area | Socioeconomic deprivation (by school type) | Selection | Acceptance | Negative^b)^ | Medicine | Fair access to medicine? Retrospective analysis of UK medical schools application data 2009–2012 using three measures of socioeconomic status | Applicants from independent and grammar schools had higher than average accepted offer ratios, and they were more likely to come from affluent postcodes. | The United Kingdom | Steven et al. [6] (2016) |
| 10 | Geographic area | Socioeconomic deprivation (by school type) | Selection | Offer of a place | Not significant^b)^ | Medicine | Evaluation of the effect of socio-economic status on performance in a Multiple Mini Interview for admission to medical school | The offer rate was slightly higher for applicants from non-selective, non-fee-paying schools than for applicants from selective and/or fee-paying schools, but not statistically significant. | The United Kingdom | Taylor et al. [7] (2014) |
| 11 | Geographic area | Socioeconomic deprivation (by POLAR3) | Selection | Offer of a place | Not significant^b)^ | Medicine | Evaluation of the effect of socio-economic status on performance in a Multiple Mini Interview for admission to medical school | Using POLAR 3 quantiles (which represent the probability that a randomly selected young person from the applicants’ area would participate in higher education), applicants from areas with lower participation rate (lower POLAR3 quantiles) were more likely to receive an offer. | The United Kingdom | Taylor et al. [7] (2014) |
| 12 | Geographic area | Socioeconomic deprivation (by school catchment) | Selection | Offer of a place | Not significant | Medicine | The BioMedical Admissions Test for medical student selection: issues of fairness and bias | Neighborhood deprivation does not have a significant effect on the likelihood of receiving an offer of a place in medical school. | The United Kingdom | Emery et al. [8] (2010) |
| 13 | Geographic area | Socioeconomic deprivation (by home residence) | Selection | Offer of a place | Not significant | Medicine | Applicant characteristics and their influence on success: results from an analysis of applicants to the University of Adelaide Medical School, 2004–2007 | Socioeconomic deprivation (by home residence) has a mixed effect on the likelihood of receiving an offer of a place in medical school.  Applicants from the lowest SES areas (home IRSD Q1) had higher odds of receiving an offer of a place compared with those from Q2, but no significant differences in several other quartile comparisons. However, applicants from Q2 had lower odds of receiving an offer of a place compared to Q4 (most advantaged area). | Australia | Laurence et al. [9] (2010) |
| 14 | Geographic area | Socioeconomic deprivation (by school catchment) | Selection | Offer of a place | Not significant^c)^ | Medicine | Applicant characteristics and their influence on success: results from an analysis of applicants to the University of Adelaide Medical School, 2004–2007 | For school IRSD, differences in offer rates between quartiles were generally not statistically significant. | Australia | Laurence et al. [9] (2010) |
| 15 | Geographic area | Socioeconomic deprivation (by home residence) | Selection | Offer of a place | Not significant^b)^ | Medicine | Assessment of personal qualities in relation to admission to medical school | Socioeconomic deprivation (by home residence) does not have a statistically significant effect on the likelihood of receiving an offer of a place in medical school. | The United Kingdom (Scotland) | Lumsden et al. [10] (2005) |
| 16 | Geographic area | Socioeconomic deprivation (by home residence) | Selection | Offer of a place | Negative | Medicine | Are efforts to attract graduate applicants to UK medical schools effective in increasing the participation of under-represented socioeconomic groups? A national cohort study | Odds of receiving an offer decline progressively with increasing area deprivation. | The United Kingdom | Kumwenda et al. [11] (2018) |
| 17 | Household economic and educational disadvantage composite^a)^ | Parental income | Selection | Enrolment | Negative^b)^ | Medicine | Temporal trends in childhood household income among applicants and matriculants to medical school and the likelihood of acceptance by income, 2014–2019 | Lower parental income was associated with lower likelihood of acceptance into an MD program. | The United States | Nguyen et al. [12] (2023) |
| 18 | Household economic and educational disadvantage composite^a)^ | Parental income | Selection | Acceptance | Negative | Medicine | Association of applicant demographic factors with medical school acceptance | Applicants from low-income parental backgrounds were underrepresented among accepted applicants. | The United States | Perez et al. [13] (2023) |
| 19 | Household economic and educational disadvantage composite^a)^ | Parental income | Selection | Acceptance | Negative | Medicine | The impact of socioeconomic factors on medical school acceptance rates | Low family income has a negative effect on medical school acceptance. | The United States | C. Williams et al. [14] (2021) |
| 20 | Household economic and educational disadvantage composite^a)^ | Parental income | Selection | Acceptance | Negative | Medicine | Sociodemographic factors and research experience impact MD-PhD program acceptance | Applicants with the lowest parental income were less likely to be accepted than those in the highest income group. | The United States | D.K.A. Williams et al. [15] (2024) |
| 21 | Household economic and educational disadvantage composite^a)^ | Parental occupation | Selection | Acceptance | Negative | Medicine | Fair access to medicine? Retrospective analysis of UK medical schools application data 2009–2012 using three measures of socioeconomic status | Students with socioeconomically disadvantaged parental occupation had lower odds of acceptance into the program | The United Kingdom | Steven et al. [6] (2016) |
| 22 | Household economic and educational disadvantage composite^a)^ | Parental occupation | Selection | Offer of a place | Negative | Medicine | Are efforts to attract graduate applicants to UK medical schools effective in increasing the participation of under-represented socioeconomic groups? A national cohort study | Students with socioeconomically disadvantaged parental occupation had lower odds of receiving an offer. | The United Kingdom | Kumwenda et al. [11] (2018) |
| 23 | Household economic and educational disadvantage composite^a)^ | Parental occupation | Selection | Offer of a place | Negative | Medicine | Population-based longitudinal analyses of offer likelihood in UK medical schools: 1996–2012 | Students with socioeconomically disadvantaged parental occupation had lower odds of receiving an offer. | The United Kingdom | Mathers et al. [16] (2016a) |
| 24 | Household economic and educational disadvantage composite^a)^ | Parental occupation | Selection | Offer of a place | Negative | Medicine | Longitudinal assessment of the impact of the use of the UK clinical aptitude test for medical student selection | Students with socioeconomically disadvantaged parental occupation had lower odds of receiving an offer. | The United Kingdom | Mathers et al. [17] (2016b) |
| 25 | Household economic and educational disadvantage composite^a)^ | Parental occupation | Selection | Offer of a place | Negative | Medicine | Widening access to UK medical education for under-represented socioeconomic groups: modelling the impact of the UKCAT in the 2009 cohort | Students with socioeconomically disadvantaged parental occupation had lower odds of receiving an offer. | The United Kingdom | Tiffin et al. [18] (2012) |
| 26 | Household economic and educational disadvantage composite^a)^ | Parental education (first generation university applicant) | Selection | Acceptance | Negative | Medicine | Association of applicant demographic factors with medical school acceptance | First-generation applicants are underrepresented among accepted applicants compared to their proportion in all applicants. | The United States | Perez et al. [13] (2023) |
| 27 | Household economic and educational disadvantage composite^a)^ | Parental education (first generation university applicant) | Selection | Acceptance | Negative | Medicine | The Impact of socioeconomic factors on medical school acceptance rates | First-generation applicant status has a negative effect on medical school acceptance. | The United States | C. Williams et al. [14] (2021) |
| 28 | Household economic and educational disadvantage composite^a)^ | Parental education (first generation university applicant) | Selection | Acceptance | Negative | Medicine | Sociodemographic factors and research experience impact MD-PhD program acceptance | Students with no parent holding a bachelor’s degree (“first-generation university applicants”) had lower odds of acceptance. | The United States | D.K.A. Williams et al. [15] (2024) |
| 29 | Household economic and educational disadvantage composite^a)^ | Parental education (first generation university applicant) | Selection | Acceptance | Not significant | Medicine | Ethnic and social disparities in performance on medical school selection criteria | Parental education (first-generation university applicant status) does not have a statistically significant effect on the likelihood of acceptance into medical school. | Netherlands | Stegers-Jager et al. [19] (2014) |
| 30 | Household economic and educational disadvantage composite^a)^ | Parental education | Selection | Acceptance | Negative^b)^ | Medicine | Social inequality in admission chances for prestigious higher education programs in Germany: do application patterns matter? | Applicants with no college-educated parents (low-SES applicants) were less likely to be accepted than those with two college-educated parents. | Germany | Finger et al. [20] (2024) |
| 31 | Household economic and educational disadvantage composite^a)^ | Education-occupation (EO) | Selection | Acceptance | Negative | Medicine | Exploring institutional stratification: minority‐serving institutional pathways to medical school acceptance in the United States | Being from low SES households (parents with EO1-EO2) has a negative effect on the likelihood of acceptance to medical school. | The United States | Burbage & Hewitt [21] (2024) |
| 32 | Household economic and educational disadvantage composite^a)^ | Education-occupation (EO) | Selection | Enrolment | Not significant | Medicine | An equivalence study of interview platform: does videoconference technology impact medical school acceptance rates of different groups? | There was no significant difference in the unadjusted odds of acceptance between applicants from low SES households (i.e., with parents in EO1–EO2 categories) and those from higher SES households. | The United States | Ballejos et al. [22] (2018) |
| 33 | Household economic and educational disadvantage composite^a)^ | Self-identified disadvantaged | Selection | Acceptance | Not significant^c)^ | Medicine | An equivalence study of interview platform: does videoconference technology impact medical school acceptance rates of different groups? | There was no significant difference in the unadjusted odds of acceptance between students who self-identified as disadvantaged and those who did not. | The United States | Ballejos et al. [22] (2018) |
| 34 | Household economic and educational disadvantage composite^a)^ | Self-identified disadvantaged | Selection | Acceptance | Negative | Medicine | Self-Reported disadvantage in medical school admissions: a call to review, revise, and further advance holistic review | Self-identified disadvantaged applicants have lower acceptance and matriculation rates. | The United States | Harrison et al. [23] (2023) |
| 35 | Household economic and educational disadvantage composite^a)^ | Self-identified disadvantaged | Selection | Offer of a place | Positive | Positive^b)^ | How medical school applicant race, ethnicity, and socioeconomic status relate to multiple mini-interview–based admissions outcomes | Lower SES is associated with higher odds of being recommended for acceptance. | The United States | Jerant et al. [24] (2015) |
| 36 | Household economic and educational disadvantage composite^a)^ | Socioeconomic group | Selection | Acceptance | Negative | Medicine | Medical school admissions across socioeconomic groups: an analysis across race neutral and race sensitive admissions cycles | The odds of being accepted was lower for the applicant in the lowest socioeconomic group. | The United States | Kennedy [25] (2010) |

POLAR3: participation of local areas, is based on the probability that a randomly selected young person from the applicants’ area would participate in higher education. EO: determination of an EO indicator is based on having both education and occupation information for at least one parent. When an applicant has complete information for two or more parents, the EO indicator for that applicant is based on the highest value among all parents [26].

^a)^Household Economic and Educational Disadvantage Composite encompasses parental income, parental occupation, and parental education, as well as cases where a combination (composite) of these indicators is used to define disadvantage. ^b)^Not included in the meta-analysis due to insufficient or unsuitable data for effect size calculation. ^c)^Not included in the meta-analysis due to overlapping study populations with another included study, which would result in double-counting of participants.

References

1. Bagg W, Curtis E, Eggleton KS, Nixon G, Bristowe Z, Brunton P, Hendry C, Kool B, Scarf D, Shaw S, Tukuitonga C, Williman J, Wilson D, Crampton P. Socio-demographic profile of medical students in Aotearoa, New Zealand (2016-2020): a nationwide cross-sectional study. BMJ Open 2023;13:e073996. <https://doi.org/10.1136/bmjopen-2023-073996>

2. Griffin B, Hu W. The interaction of socio-economic status and gender in widening participation in medicine. Med Educ 2015;49:103-113. <https://doi.org/10.1111/medu.12480>

3. James D, Ferguson E, Powis D, Symonds I, Yates J. Graduate entry to medicine: widening academic and socio-demographic access. Med Educ 2008;42:294-300. <https://doi.org/10.1111/j.1365-2923.2008.03006.x>

4. Zhang D, Li G, Mu L, Thapa J, Li Y, Chen Z, Shi L, Su D, Son H, Pagan JA. Trends in medical school application and matriculation rates across the United States from 2001 to 2015: implications for health disparities. Acad Med 2021;96:885-893. <https://doi.org/10.1097/ACM.0000000000004033>

5. Pitre T, Thomas A, Evans K, Jones A, Mountjoy M, Costa AP. The influence of income on medical school admissions in Canada: a retrospective cohort study. BMC Med Educ 2020;20:209. <https://doi.org/10.1186/s12909-020-02126-0>

6. Steven K, Dowell J, Jackson C, Guthrie B. Fair access to medicine?: retrospective analysis of UK medical schools application data 2009-2012 using three measures of socioeconomic status. BMC Med Educ 2016;16:11. <https://doi.org/10.1186/s12909-016-0536-1>

7. Taylor CA, Green KE, Spruce A. Evaluation of the effect of socio-economic status on performance in a Multiple Mini Interview for admission to medical school. Med Teach 2015;37:59-63. <https://doi.org/10.3109/0142159X.2014.923562>

8. Emery JL, Bell JF, Vidal Rodeiro CL. The BioMedical Admissions Test for medical student selection: issues of fairness and bias. Med Teach 2011;33:62-71. <https://doi.org/10.3109/0142159X.2010.528811>

9. Laurence CO, Turnbull DA, Briggs NE, Robinson JS. Applicant characteristics and their influence on success: results from an analysis of applicants to the University of Adelaide Medical School, 2004-2007. Med J Aust 2010;192:212-216. <https://doi.org/10.5694/j.1326-5377.2010.tb03481.x>

10. Lumsden MA, Bore M, Millar K, Jack R, Powis D. Assessment of personal qualities in relation to admission to medical school. Med Educ 2005;39:258-265. <https://doi.org/10.1111/j.1365-2929.2005.02087.x>

11. Kumwenda B, Cleland J, Greatrix R, MacKenzie RK, Prescott G. Are efforts to attract graduate applicants to UK medical schools effective in increasing the participation of under-represented socioeconomic groups?: a national cohort study. BMJ Open 2018;8:e018946. <https://doi.org/10.1136/bmjopen-2017-018946>

12. Nguyen M, Desai MM, Fancher TL, Chaudhry SI, Mason HR, Boatright D. Temporal trends in childhood household income among applicants and matriculants to medical school and the likelihood of acceptance by income, 2014-2019. JAMA 2023;329:1882-1884. <https://doi.org/10.1001/jama.2023.5654>

13. Perez MA, Williams C, Henderson K, McGregor R, Vapiwala N, Shea JA, Dine CJ. Association of applicant demographic factors with medical school acceptance. BMC Med Educ 2023;23:960. <https://doi.org/10.1186/s12909-023-04897-8>

14. Williams C, Perez MA, Vapiwala N, Shea JA. The impact of socioeconomic factors on medical school acceptance rates. Acad Med 2021;96:S219-S220. <https://doi.org/10.1097/ACM.0000000000004281>

15. Williams DK, Christophers B, Keyes T, Kumar R, Granovetter MC, Adigun A, Olivera J, Pura-Bryant J, Smith C, Okafor C, Shibre M, Daye D, Akabas MH. Sociodemographic factors and research experience impact MD-PhD program acceptance. JCI Insight 2024;9:e176146. <https://doi.org/10.1172/jci.insight.176146>

16. Mathers J, Sitch A, Parry J. Population-based longitudinal analyses of offer likelihood in UK medical schools: 1996-2012. Med Educ 2016;50:612-623. <https://doi.org/10.1111/medu.12981>

17. Mathers J, Sitch A, Parry J. Longitudinal assessment of the impact of the use of the UK clinical aptitude test for medical student selection. Med Educ 2016;50:1033-1044. <https://doi.org/10.1111/medu.13082>

18. Tiffin PA, Dowell JS, McLachlan JC. Widening access to UK medical education for under-represented socioeconomic groups: modelling the impact of the UKCAT in the 2009 cohort. BMJ 2012;344:e1805. <https://doi.org/10.1136/bmj.e1805>

19. Stegers-Jager KM, Steyerberg EW, Lucieer SM, Themmen AP. Ethnic and social disparities in performance on medical school selection criteria. Med Educ 2015;49:124-133. <https://doi.org/10.1111/medu.12536>

20. Finger C, Solga H, Elbers B. Social inequality in admission chances for prestigious higher education programs in Germany: do application patterns matter? Eur Sociol Rev 2024;40:1013-1029. <https://doi.org/10.1093/esr/jcae024>

21. Burbage AK, Hewitt EY. Exploring institutional stratification: minority-serving institutional pathways to medical school acceptance in the United States. Med Educ 2025;59:640-651. <https://doi.org/10.1111/medu.15539>

22. Ballejos MP, Oglesbee S, Hettema J, Sapien R. An equivalence study of interview platform: does videoconference technology impact medical school acceptance rates of different groups? Adv Health Sci Educ Theory Pract 2018;23:601-610. <https://doi.org/10.1007/s10459-018-9817-2>

23. Harrison LE, Fletcher L, Dunleavy D, Price-Johnson T, Vashi Kundu R, Fogerty GT, Berardi-Demo L. Self-reported disadvantage in medical school admissions: a call to review, revise, and further advance holistic review. Acad Med 2023;98:1044-1052. <https://doi.org/10.1097/ACM.0000000000005272>

24. Jerant A, Fancher T, Fenton JJ, Fiscella K, Sousa F, Franks P, Henderson M. How medical school applicant race, ethnicity, and socioeconomic status relate to multiple mini-interview-based admissions outcomes: findings from one medical school. Acad Med 2015;90:1667-1674. <https://doi.org/10.1097/ACM.0000000000000766>

25. Kennedy M. Medical school admissions across socioeconomic groups: An analysis across race neutral and race sensitive admissions cycles [dissertation]. University of North Texas; 2010.

26. Grbic D, Jones DJ, Case ST. The role of socioeconomic status in medical school admissions: validation of a socioeconomic indicator for use in medical school admissions. Acad Med 2015;90:953-960. <https://doi.org/10.1097/ACM.0000000000000653>
