# Supplementary material for: Socioeconomic and economic factors affecting access and progression in medical schools: a systematic review and meta-analysis
Source: J Educ Eval Health Prof. 2026 Apr 16;23:6. doi: 10.3352/jeehp.2026.23.6 (PMC13181141; doi:10.3352/jeehp.2026.23.6)
Supplement: Supplementary file 7 — Supplement 5. Impact of economic factors on students’ progression in medicine and dentistry programs. [file jeehp-23-06-suppl5.docx]

**Supplement 5.** Impact of economic factors on students' progression in medicine programs

| No. | Category of economic factor | Predictor | Category of outcome | Outcome | Effect direction | Field | Title | Summary | Country | Author |
| --- | --- | --- | --- | --- | --- | --- | --- | --- | --- | --- |
| 1 | Debt | Premedical debt | Progression | Retention | Negative | Medicine | Prematriculation variables associated with suboptimal outcomes for the 1994-1999 cohort of us medical school matriculants | Higher premedical debt is associated with higher odds of both academic and non-academic withdrawal/dismissal. | The United States | Andriole & Jeffe [1] (2010) |
| 2 | Debt | Premedical debt | Progression | Retention | Not significant | Medicine | Educational outcomes for students enrolled in MD–PhD programs at medical school matriculation, 1995–2000 | Premedical debt does not have a statistically significant effect on withdrawal/dismissal from medical school in this MD–PhD cohort. | The United States | Jeffe et al. [2] (2014) |
| 3 | Household economic and educational disadvantage composite^a)^ | Parental income | Progression | Graduation | Negative | Medicine | First-generation and low-income students in the national medical student body | Students from lowest parental income groups had lower graduation probabilities compared with students in the highest income group. | The United States | Kamran et al. [3] (2025) |
| 4 | Household economic and educational disadvantage composite^a)^ | Parental income | Progression | Success | Not significant | Medicine | Factors associated with academic success at Vienna Medical School: prospective survey | The parents’ monthly income had no significant influence on students’ success. | Austria | Frischenschlager et al. [4] (2005) |
| 5 | Household economic and educational disadvantage composite^a)^ | Parental income | Progression | Retention | Negative | Medicine | Association of sociodemographic characteristics with US medical student attrition | Students from low-income parental backgrounds have higher attrition rates. | The United States | Nguyen et al. [5] (2022) |
| 6 | Household economic and educational disadvantage composite^a)^ | Parental education (first generation university applicant) | Progression | Success | Negative^b)^ | Medicine | Risk factors associated with academic difficulty in an Australian regionally located medical school | Students who were first in their family to go to university were more likely to experience academic difficulty than those who were not. | Australia | Malau-Aduli et al. [6] (2017) |
| 7 | Household economic and educational disadvantage composite^a)^ | Parental education (first generation university applicant) | Progression | Success | Negative | Medicine | Factors associated with academic success at Vienna Medical School: prospective survey | Students whose fathers^c)^ had not attended university (“first-generation university applicants”) had lower odds of success than their counterparts. | Austria | Frischenschlager et al. [4] (2005) |
| 8 | Household economic and educational disadvantage composite^a)^ | Parental education (first generation university applicant) | Progression | Success | Negative | Medicine | The validity of MCAT scores in predicting students’ performance and progress in medical school: results from a multisite study | Students with no parent holding a bachelor’s degree (“first-generation university applicants”) had lower odds of success (defined as completion of the coursework on time or within one extra year) than those with at least one parent holding a bachelor’s degree | Canada and The United States | Hanson et al. [7] (2022) |
| 9 | Household economic and educational disadvantage composite^a)^ | Parental education (first generation university applicant) | Progression | Graduation | Negative | Medicine | First-generation and low-income students in the national medical student body | First-generation students have lower odds of graduation. | The United States | Kamran et al. [3] (2025) |
| 10 | Geographic area | Socioeconomic deprivation (by home residence) | Progression | Success | Negative^b)^ | Medicine | Risk factors associated with academic difficulty in an Australian regionally located medical school | Students from very remote areas were more likely to experience academic difficulty than their counterparts | Australia | Malau-Aduli et al. [6] (2017) |
| 11 | Geographic area | Socioeconomic deprivation (by home residence) | Progression | Retention | Negative | Medicine | Association of sociodemographic characteristics with US medical student attrition | Students from underresourced neighborhoods had higher attrition rates. | The United States | Nguyen et al. [5] (2022) |

^a)^Household economic and educational disadvantage composite encompasses parental income, parental occupation, and parental education, as well as cases where a combination (composite) of these indicators is used to define disadvantage. ^b)^Not included in the meta-analysis due to insufficient or unsuitable data for effect size calculation. ^c)^In this study, data on mother’s educational attainment were also reported separately, but were not included in the calculation of this effect size.

**References**

1. Andriole DA, Jeffe DB. Prematriculation variables associated with suboptimal outcomes for the 1994-1999 cohort of US medical school matriculants. JAMA 2010;304:1212-1219. <https://doi.org/10.1001/jama.2010.1321>

2. Jeffe DB, Andriole DA, Wathington HD, Tai RH. Educational outcomes for students enrolled in MD-PhD programs at medical school matriculation, 1995-2000: a national cohort study. Acad Med 2014;89:84-93. <https://doi.org/10.1097/ACM.0000000000000071>

3. Kamran SC, Pompa IR, Nguyen HB, Cha J, Salinas KE, Niemierko A, Vapiwala N. First-generation and low-income students in the national medical student body. JAMA Netw Open 2025;8:e259769. <https://doi.org/10.1001/jamanetworkopen.2025.9769>

4. Frischenschlager O, Haidinger G, Mitterauer L. Factors associated with academic success at Vienna Medical School: prospective survey. Croat Med J 2005;46:58-65.

5. Nguyen M, Chaudhry SI, Desai MM, Chen C, Mason HR, McDade WA, Fancher TL, Boatright D. Association of sociodemographic characteristics with US medical student attrition. JAMA Intern Med 2022;182:917-924. <https://doi.org/10.1001/jamainternmed.2022.2194>

6. Malau-Aduli BS, O’Connor T, Ray RA, van der Kruk Y, Bellingan M, Teague PA. Risk factors associated with academic difficulty in an Australian regionally located medical school. BMC Med Educ 2017;17:266. <https://doi.org/10.1186/s12909-017-1095-9>

7. Hanson JT, Busche K, Elks ML, Jackson-Williams LE, Liotta RA, Miller C, Morris CA, Thiessen B, Yuan K. The validity of MCAT scores in predicting students’ performance and progress in medical school: results from a multisite study. Acad Med 2022;97:1374-1384. <https://doi.org/10.1097/ACM.0000000000004754>
