# Supplementary material for: Socioeconomic and economic factors affecting access and progression in medical schools: a systematic review and meta-analysis
Source: J Educ Eval Health Prof. 2026 Apr 16;23:6. doi: 10.3352/jeehp.2026.23.6 (PMC13181141; doi:10.3352/jeehp.2026.23.6)
Supplement: Supplementary file 8 — Supplement 6. Funnel plot. [file jeehp-23-06-suppl6.docx]

**Supplement 6.** Funnel plot


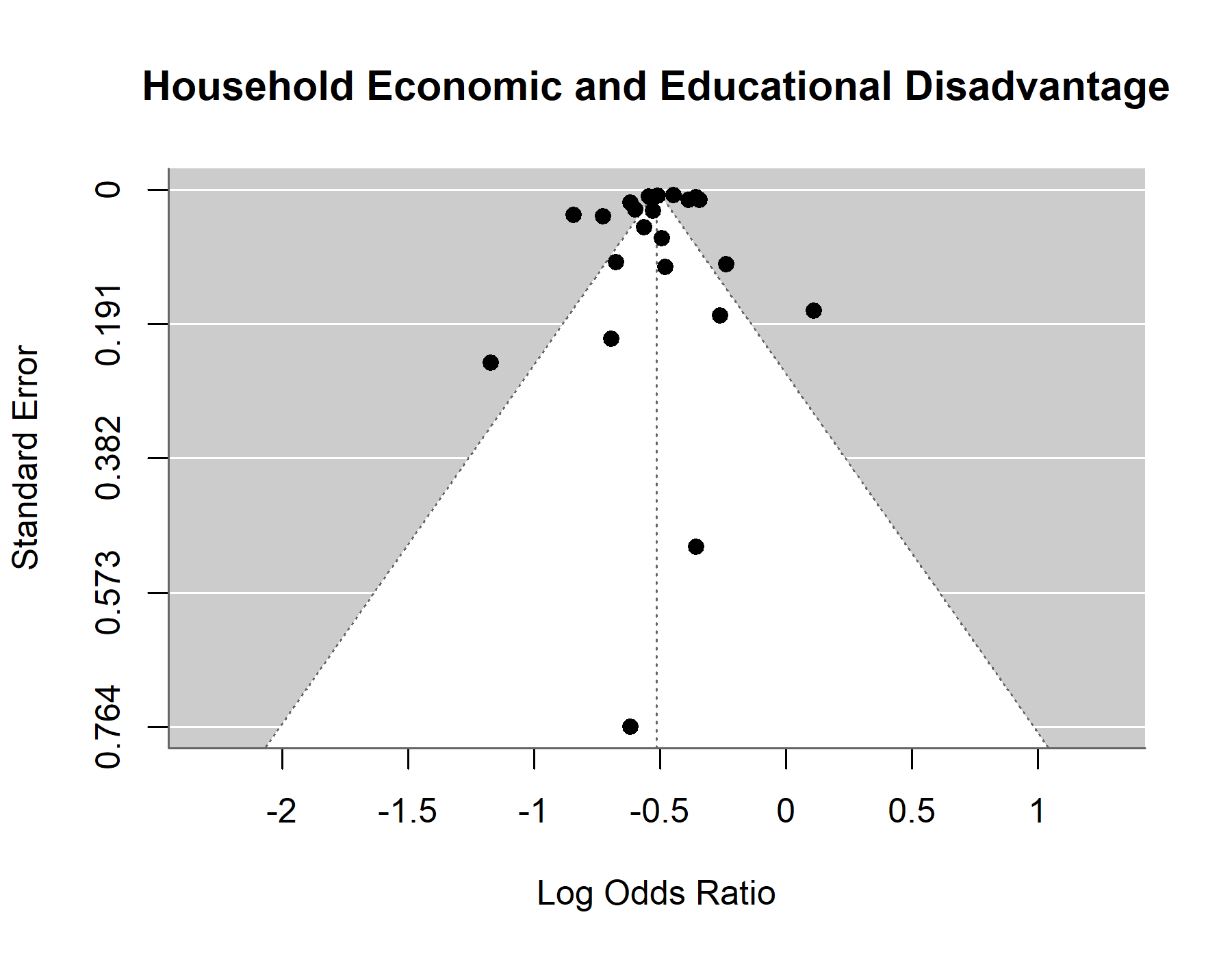


Funnel plot assessing publication bias for the composite predictor of household economic and educational disadvantage on medical school selection. The plot displays the distribution of the extracted effect sizes (log odds ratio) against their corresponding standard errors for the 22 included studies. The visually symmetrical scatter of data points around the central pooled estimate suggests an absence of small-study effects. Egger’s regression test confirmed no significant asymmetry (z=0.075, P=0.940), indicating no statistical evidence of publication bias within this primary analysis.
