# Supplementary material for: Socioeconomic and economic factors affecting access and progression in medical schools: a systematic review and meta-analysis
Source: J Educ Eval Health Prof. 2026 Apr 16;23:6. doi: 10.3352/jeehp.2026.23.6 (PMC13181141; doi:10.3352/jeehp.2026.23.6)
Supplement: Supplementary file 9 — Supplement 7. Bias appraisal. [file jeehp-23-06-suppl7.docx]

**Supplement 7.** Bias appraisal

| Article | 1. Is the source population representative of the population of interest? | 2. Is the response rate adequate? | 3. Is there little missing data?^a)^ | 4. Is the survey clinically sensible? | 5. Is there any evidence for the reliability and validity of the survey instrument? |
| --- | --- | --- | --- | --- | --- |
| [1] Andriole DA, Jeffe DB. Prematriculation variables associated with suboptimal outcomes for the 1994-1999 cohort of US medical school matriculants. JAMA 2010;304:1212-1219. <https://doi.org/10.1001/jama.2010.1321> | Definitely yes (low risk of bias) | NA^b)^ | Definitely yes (low risk of bias) | NA^c)^ | NA^d)^ |
| [2] Bagg W, Curtis E, Eggleton KS, Nixon G, Bristowe Z, Brunton P, Hendry C, Kool B, Scarf D, Shaw S, Tukuitonga C, Williman J, Wilson D, Crampton P. Socio-demographic profile of medical students in Aotearoa, New Zealand (2016-2020): a nationwide cross-sectional study. BMJ Open 2023;13:e073996. <https://doi.org/10.1136/bmjopen-2023-073996> | Definitely yes (low risk of bias) | NA^b)^ | Definitely yes (low risk of bias) | NA^c)^ | NA^d)^ |
| [3] Ballejos MP, Oglesbee S, Hettema J, Sapien R. An equivalence study of interview platform: does videoconference technology impact medical school acceptance rates of different groups? Adv Health Sci Educ Theory Pract 2018;23:601-610. <https://doi.org/10.1007/s10459-018-9817-2> | Probably yes | NA^b)^ | Definitely yes (low risk of bias) | NA^c)^ | NA^d)^ |
| [4] Burbage AK, Hewitt EY. Exploring institutional stratification: minority-serving institutional pathways to medical school acceptance in the United States. Med Educ 2025;59:640-651. <https://doi.org/10.1111/medu.15539> | Definitely yes (low risk of bias) | NA^b)^ | Definitely yes (low risk of bias) | NA^c)^ | NA^d)^ |
| [5] Emery JL, Bell JF, Vidal Rodeiro CL. The BioMedical Admissions Test for medical student selection: issues of fairness and bias. Med Teach 2011;33:62-71. <https://doi.org/10.3109/0142159X.2010.528811> | Probably yes | NA^b)^ | Definitely yes (low risk of bias) | NA^c)^ | NA^d)^ |
| [6] Finger C, Solga H, Elbers B. Social inequality in admission chances for prestigious higher education programs in Germany: do application patterns matter? Eur Sociol Rev 2024;40:1013-1029. <https://doi.org/10.1093/esr/jcae024> | Definitely yes (low risk of bias) | NA^b)^ | Definitely yes (low risk of bias) | NA^c)^ | NA^d)^ |
| [7] Frischenschlager O, Haidinger G, Mitterauer L. Factors associated with academic success at Vienna Medical School: prospective survey. Croat Med J 2005;46:58-65. | Probably yes | NA^b)^ | Probably no | NA^c)^ | NA^d)^ |
| [8] Griffin B, Hu W. The interaction of socio-economic status and gender in widening participation in medicine. Med Educ 2015;49:103-113. <https://doi.org/10.1111/medu.12480> | Probably yes | NA^b)^ | Definitely yes (low risk of bias) | NA^c)^ | NA^d)^ |
| [9] Hanson JT, Busche K, Elks ML, Jackson-Williams LE, Liotta RA, Miller C, Morris CA, Thiessen B, Yuan K. The validity of MCAT scores in predicting students’ performance and progress in medical school: results from a multisite study. Acad Med 2022;97:1374-1384. <https://doi.org/10.1097/ACM.0000000000004754> | Definitely yes (low risk of bias) | NA^b)^ | Definitely yes (low risk of bias) | NA^c)^ | NA^d)^ |
| [10] Harrison LE, Fletcher L, Dunleavy D, Price-Johnson T, Vashi Kundu R, Fogerty GT, Berardi-Demo L. Self-reported disadvantage in medical school admissions: a call to review, revise, and further advance holistic review. Acad Med 2023;98:1044-1052. <https://doi.org/10.1097/ACM.0000000000005272> | Definitely yes (low risk of bias) | NA^b)^ | Probably yes | NA^c)^ | NA^d)^ |
| [11] James D, Ferguson E, Powis D, Symonds I, Yates J. Graduate entry to medicine: widening academic and socio-demographic access. Med Educ 2008;42:294-300. <https://doi.org/10.1111/j.1365-2923.2008.03006.x> | Probably yes | NA^b)^ | Definitely yes (low risk of bias) | NA^c)^ | NA^d)^ |
| [12] Jeffe DB, Andriole DA, Wathington HD, Tai RH. Educational outcomes for students enrolled in MD-PhD programs at medical school matriculation, 1995-2000: a national cohort study. Acad Med 2014;89:84-93. <https://doi.org/10.1097/ACM.0000000000000071> | Definitely yes (low risk of bias) | NA^b)^ | Definitely yes (low risk of bias) | NA^c)^ | NA^d)^ |
| [13] Jerant A, Fancher T, Fenton JJ, Fiscella K, Sousa F, Franks P, Henderson M. How medical school applicant race, ethnicity, and socioeconomic status relate to multiple mini-interview-based admissions outcomes: findings from one medical school. Acad Med 2015;90:1667-1674. <https://doi.org/10.1097/ACM.0000000000000766> | Probably yes | NA^b)^ | Definitely yes (low risk of bias) | NA^c)^ | NA^d)^ |
| [14] Kamran SC, Pompa IR, Nguyen HB, Cha J, Salinas KE, Niemierko A, Vapiwala N. First-generation and low-income students in the national medical student body. JAMA Netw Open 2025;8:e259769. <https://doi.org/10.1001/jamanetworkopen.2025.9769> | Definitely yes (low risk of bias) | NA^b)^ | Definitely yes (low risk of bias) | NA^c)^ | NA^d)^ |
| [15] Kennedy M. Medical school admissions across socioeconomic groups: An analysis across race neutral and race sensitive admissions cycles [dissertation]. University of North Texas; 2010. | Probably yes | NA^b)^ | Probably yes | NA^c)^ | NA^d)^ |
| [16] Kumwenda B, Cleland J, Greatrix R, MacKenzie RK, Prescott G. Are efforts to attract graduate applicants to UK medical schools effective in increasing the participation of under-represented socioeconomic groups?: a national cohort study. BMJ Open 2018;8:e018946. <https://doi.org/10.1136/bmjopen-2017-018946> | Definitely yes (low risk of bias) | NA^b)^ | Definitely yes (low risk of bias) | NA^c)^ | NA^d)^ |
| [17] Laurence CO, Turnbull DA, Briggs NE, Robinson JS. Applicant characteristics and their influence on success: results from an analysis of applicants to the University of Adelaide Medical School, 2004-2007. Med J Aust 2010;192:212-216. <https://doi.org/10.5694/j.1326-5377.2010.tb03481.x> | Probably yes | NA^b)^ | Definitely yes (low risk of bias) | NA^c)^ | NA^d)^ |
| [18] Lumsden MA, Bore M, Millar K, Jack R, Powis D. Assessment of personal qualities in relation to admission to medical school. Med Educ 2005;39:258-265. <https://doi.org/10.1111/j.1365-2929.2005.02087.x> | Definitely yes (low risk of bias) | NA^b)^ | Definitely yes (low risk of bias) | NA^c)^ | NA^d)^ |
| [19] Malau-Aduli BS, O’Connor T, Ray RA, van der Kruk Y, Bellingan M, Teague PA. Risk factors associated with academic difficulty in an Australian regionally located medical school. BMC Med Educ 2017;17:266. <https://doi.org/10.1186/s12909-017-1095-9> | Probably yes | NA^b)^ | Definitely yes (low risk of bias) | NA^c)^ | NA^d)^ |
| [20] Mathers J, Sitch A, Parry J. Longitudinal assessment of the impact of the use of the UK clinical aptitude test for medical student selection. Med Educ 2016;50:1033-1044. <https://doi.org/10.1111/medu.13082> | Definitely yes (low risk of bias) | NA^b)^ | Definitely yes (low risk of bias) | NA^c)^ | NA^d)^ |
| [21] Mathers J, Sitch A, Parry J. Population-based longitudinal analyses of offer likelihood in UK medical schools: 1996-2012. Med Educ 2016;50:612-623. <https://doi.org/10.1111/medu.12981> | Definitely yes (low risk of bias) | NA^b)^ | Definitely yes (low risk of bias) | NA^c)^ | NA^d)^ |
| [22] Nguyen M, Chaudhry SI, Desai MM, Chen C, Mason HR, McDade WA, Fancher TL, Boatright D. Association of sociodemographic characteristics with US medical student attrition. JAMA Intern Med 2022;182:917-924. <https://doi.org/10.1001/jamainternmed.2022.2194> | Definitely yes (low risk of bias) | NA^b)^ | Definitely yes (low risk of bias) | NA^c)^ | NA^d)^ |
| [23] Nguyen M, Desai MM, Fancher TL, Chaudhry SI, Mason HR, Boatright D. Temporal trends in childhood household income among applicants and matriculants to medical school and the likelihood of acceptance by income, 2014-2019. JAMA 2023;329:1882-1884. <https://doi.org/10.1001/jama.2023.5654> | Definitely yes (low risk of bias) | NA^b)^ | Definitely yes (low risk of bias) | NA^c)^ | NA^d)^ |
| [24] Perez MA, Williams C, Henderson K, McGregor R, Vapiwala N, Shea JA, Dine CJ. Association of applicant demographic factors with medical school acceptance. BMC Med Educ 2023;23:960. <https://doi.org/10.1186/s12909-023-04897-8> | Definitely yes (low risk of bias) | NA^b)^ | Definitely yes (low risk of bias) | NA^c)^ | NA^d)^ |
| [25] Pitre T, Thomas A, Evans K, Jones A, Mountjoy M, Costa AP. The influence of income on medical school admissions in Canada: a retrospective cohort study. BMC Med Educ 2020;20:209. <https://doi.org/10.1186/s12909-020-02126-0> | Probably yes | NA^b)^ | Probably yes | NA^c)^ | NA^d)^ |
| [26] Stegers-Jager KM, Steyerberg EW, Lucieer SM, Themmen AP. Ethnic and social disparities in performance on medical school selection criteria. Med Educ 2015;49:124-133. <https://doi.org/10.1111/medu.12536> | Probably yes | NA^b)^ | Definitely yes (low risk of bias) | NA^c)^ | NA^d)^ |
| [27] Steven K, Dowell J, Jackson C, Guthrie B. Fair access to medicine?: retrospective analysis of UK medical schools application data 2009-2012 using three measures of socioeconomic status. BMC Med Educ 2016;16:11. <https://doi.org/10.1186/s12909-016-0536-1> | Definitely yes (low risk of bias) | NA^b)^ | Definitely yes (low risk of bias) | NA^c)^ | NA^d)^ |
| [28] Taylor CA, Green KE, Spruce A. Evaluation of the effect of socio-economic status on performance in a Multiple Mini Interview for admission to medical school. Med Teach 2015;37:59-63. <https://doi.org/10.3109/0142159X.2014.923562> | Definitely yes (low risk of bias) | NA^b)^ | Probably yes | NA^c)^ | NA^d)^ |
| [29] Tiffin PA, Dowell JS, McLachlan JC. Widening access to UK medical education for under-represented socioeconomic groups: modelling the impact of the UKCAT in the 2009 cohort. BMJ 2012;344:e1805. <https://doi.org/10.1136/bmj.e1805> | Definitely yes (low risk of bias) | NA^b)^ | Definitely yes (low risk of bias) | NA^c)^ | NA^d)^ |
| [30] Williams C, Perez MA, Vapiwala N, Shea JA. The impact of socioeconomic factors on medical school acceptance rates. Acad Med 2021;96:S219-S220. <https://doi.org/10.1097/ACM.0000000000004281> | Definitely yes (low risk of bias) | NA^b)^ | Definitely yes (low risk of bias) | NA^c)^ | NA^d)^ |
| [31] Williams DK, Christophers B, Keyes T, Kumar R, Granovetter MC, Adigun A, Olivera J, Pura-Bryant J, Smith C, Okafor C, Shibre M, Daye D, Akabas MH. Sociodemographic factors and research experience impact MD-PhD program acceptance. JCI Insight 2024;9:e176146. <https://doi.org/10.1172/jci.insight.176146> | Definitely yes (low risk of bias) | NA^b)^ | Definitely yes (low risk of bias) | NA^c)^ | NA^d)^ |
| [32] Zhang D, Li G, Mu L, Thapa J, Li Y, Chen Z, Shi L, Su D, Son H, Pagan JA. Trends in medical school application and matriculation rates across the United States from 2001 to 2015: implications for health disparities. Acad Med 2021;96:885-893. <https://doi.org/10.1097/ACM.0000000000004033> | Definitely yes (low risk of bias) | NA^b)^ | Definitely yes (low risk of bias) | NA^c)^ | NA^d)^ |

^a)^This aspect is evaluated by comparing the number of participants whose data were analysed to the original sample size. However, it should be noted that since our meta-analysis extracts the effect of socioeconomic factors on desired outcomes, this type of missing data does not affect these effect sizes. Therefore, no bias is introduced, regardless of the evaluation. ^b)^Not applicable to studies included in our meta-analysis: as these studies reported results of binary outcomes (for either selection or progression), the response rate is not relevant. ^c)^Not applicable to our meta-analysis: studies included in our meta-analysis reported the binary results of admission processes or progress through the program, using tools and measures already used in their corresponding universities, rendering individual face validities irrelevant. ^d)^Not applicable to our meta-analysis: it should be noted that our meta-analysis seeks to determine the effects of socioeconomic factors on binary selection outcomes (selected vs. not selected) or progress outcomes (progressed vs. not progressed), rather than the validity of admission tools or outcome measures in evaluating student status. Therefore, individual validity or reliability of the tools and measures does not introduce bias to our results.

**References**

1. Andriole DA, Jeffe DB. Prematriculation variables associated with suboptimal outcomes for the 1994-1999 cohort of US medical school matriculants. JAMA 2010;304:1212-1219. <https://doi.org/10.1001/jama.2010.1321>

2. Bagg W, Curtis E, Eggleton KS, Nixon G, Bristowe Z, Brunton P, Hendry C, Kool B, Scarf D, Shaw S, Tukuitonga C, Williman J, Wilson D, Crampton P. Socio-demographic profile of medical students in Aotearoa, New Zealand (2016-2020): a nationwide cross-sectional study. BMJ Open 2023;13:e073996. <https://doi.org/10.1136/bmjopen-2023-073996>

3. Ballejos MP, Oglesbee S, Hettema J, Sapien R. An equivalence study of interview platform: does videoconference technology impact medical school acceptance rates of different groups? Adv Health Sci Educ Theory Pract 2018;23:601-610. <https://doi.org/10.1007/s10459-018-9817-2>

4. Burbage AK, Hewitt EY. Exploring institutional stratification: minority-serving institutional pathways to medical school acceptance in the United States. Med Educ 2025;59:640-651. <https://doi.org/10.1111/medu.15539>

5. Emery JL, Bell JF, Vidal Rodeiro CL. The BioMedical Admissions Test for medical student selection: issues of fairness and bias. Med Teach 2011;33:62-71. <https://doi.org/10.3109/0142159X.2010.528811>

6. Finger C, Solga H, Elbers B. Social inequality in admission chances for prestigious higher education programs in Germany: do application patterns matter? Eur Sociol Rev 2024;40:1013-1029. <https://doi.org/10.1093/esr/jcae024>

7. Frischenschlager O, Haidinger G, Mitterauer L. Factors associated with academic success at Vienna Medical School: prospective survey. Croat Med J 2005;46:58-65.

8. Griffin B, Hu W. The interaction of socio-economic status and gender in widening participation in medicine. Med Educ 2015;49:103-113. <https://doi.org/10.1111/medu.12480>

9. Hanson JT, Busche K, Elks ML, Jackson-Williams LE, Liotta RA, Miller C, Morris CA, Thiessen B, Yuan K. The validity of MCAT scores in predicting students’ performance and progress in medical school: results from a multisite study. Acad Med 2022;97:1374-1384. <https://doi.org/10.1097/ACM.0000000000004754>

10. Harrison LE, Fletcher L, Dunleavy D, Price-Johnson T, Vashi Kundu R, Fogerty GT, Berardi-Demo L. Self-reported disadvantage in medical school admissions: a call to review, revise, and further advance holistic review. Acad Med 2023;98:1044-1052. <https://doi.org/10.1097/ACM.0000000000005272>

11. James D, Ferguson E, Powis D, Symonds I, Yates J. Graduate entry to medicine: widening academic and socio-demographic access. Med Educ 2008;42:294-300. <https://doi.org/10.1111/j.1365-2923.2008.03006.x>

12. Jeffe DB, Andriole DA, Wathington HD, Tai RH. Educational outcomes for students enrolled in MD-PhD programs at medical school matriculation, 1995-2000: a national cohort study. Acad Med 2014;89:84-93. <https://doi.org/10.1097/ACM.0000000000000071>

13. Jerant A, Fancher T, Fenton JJ, Fiscella K, Sousa F, Franks P, Henderson M. How medical school applicant race, ethnicity, and socioeconomic status relate to multiple mini-interview-based admissions outcomes: findings from one medical school. Acad Med 2015;90:1667-1674. <https://doi.org/10.1097/ACM.0000000000000766>

14. Kamran SC, Pompa IR, Nguyen HB, Cha J, Salinas KE, Niemierko A, Vapiwala N. First-generation and low-income students in the national medical student body. JAMA Netw Open 2025;8:e259769. <https://doi.org/10.1001/jamanetworkopen.2025.9769>

15. Kennedy M. Medical school admissions across socioeconomic groups: An analysis across race neutral and race sensitive admissions cycles [dissertation]. University of North Texas; 2010.

16. Kumwenda B, Cleland J, Greatrix R, MacKenzie RK, Prescott G. Are efforts to attract graduate applicants to UK medical schools effective in increasing the participation of under-represented socioeconomic groups?: a national cohort study. BMJ Open 2018;8:e018946. <https://doi.org/10.1136/bmjopen-2017-018946>

17. Laurence CO, Turnbull DA, Briggs NE, Robinson JS. Applicant characteristics and their influence on success: results from an analysis of applicants to the University of Adelaide Medical School, 2004-2007. Med J Aust 2010;192:212-216. <https://doi.org/10.5694/j.1326-5377.2010.tb03481.x>

18. Lumsden MA, Bore M, Millar K, Jack R, Powis D. Assessment of personal qualities in relation to admission to medical school. Med Educ 2005;39:258-265. <https://doi.org/10.1111/j.1365-2929.2005.02087.x>

19. Malau-Aduli BS, O’Connor T, Ray RA, van der Kruk Y, Bellingan M, Teague PA. Risk factors associated with academic difficulty in an Australian regionally located medical school. BMC Med Educ 2017;17:266. <https://doi.org/10.1186/s12909-017-1095-9>

20. Mathers J, Sitch A, Parry J. Longitudinal assessment of the impact of the use of the UK clinical aptitude test for medical student selection. Med Educ 2016;50:1033-1044. <https://doi.org/10.1111/medu.13082>

21. Mathers J, Sitch A, Parry J. Population-based longitudinal analyses of offer likelihood in UK medical schools: 1996-2012. Med Educ 2016;50:612-623. <https://doi.org/10.1111/medu.12981>

22. Nguyen M, Chaudhry SI, Desai MM, Chen C, Mason HR, McDade WA, Fancher TL, Boatright D. Association of sociodemographic characteristics with US medical student attrition. JAMA Intern Med 2022;182:917-924. <https://doi.org/10.1001/jamainternmed.2022.2194>

23. Nguyen M, Desai MM, Fancher TL, Chaudhry SI, Mason HR, Boatright D. Temporal trends in childhood household income among applicants and matriculants to medical school and the likelihood of acceptance by income, 2014-2019. JAMA 2023;329:1882-1884. <https://doi.org/10.1001/jama.2023.5654>

24. Perez MA, Williams C, Henderson K, McGregor R, Vapiwala N, Shea JA, Dine CJ. Association of applicant demographic factors with medical school acceptance. BMC Med Educ 2023;23:960. <https://doi.org/10.1186/s12909-023-04897-8>

25. Pitre T, Thomas A, Evans K, Jones A, Mountjoy M, Costa AP. The influence of income on medical school admissions in Canada: a retrospective cohort study. BMC Med Educ 2020;20:209. <https://doi.org/10.1186/s12909-020-02126-0>

26. Stegers-Jager KM, Steyerberg EW, Lucieer SM, Themmen AP. Ethnic and social disparities in performance on medical school selection criteria. Med Educ 2015;49:124-133. <https://doi.org/10.1111/medu.12536>

27. Steven K, Dowell J, Jackson C, Guthrie B. Fair access to medicine?: retrospective analysis of UK medical schools application data 2009-2012 using three measures of socioeconomic status. BMC Med Educ 2016;16:11. <https://doi.org/10.1186/s12909-016-0536-1>

28. Taylor CA, Green KE, Spruce A. Evaluation of the effect of socio-economic status on performance in a Multiple Mini Interview for admission to medical school. Med Teach 2015;37:59-63. <https://doi.org/10.3109/0142159X.2014.923562>

29. Tiffin PA, Dowell JS, McLachlan JC. Widening access to UK medical education for under-represented socioeconomic groups: modelling the impact of the UKCAT in the 2009 cohort. BMJ 2012;344:e1805. <https://doi.org/10.1136/bmj.e1805>

30. Williams C, Perez MA, Vapiwala N, Shea JA. The impact of socioeconomic factors on medical school acceptance rates. Acad Med 2021;96:S219-S220. <https://doi.org/10.1097/ACM.0000000000004281>

31. Williams DK, Christophers B, Keyes T, Kumar R, Granovetter MC, Adigun A, Olivera J, Pura-Bryant J, Smith C, Okafor C, Shibre M, Daye D, Akabas MH. Sociodemographic factors and research experience impact MD-PhD program acceptance. JCI Insight 2024;9:e176146. <https://doi.org/10.1172/jci.insight.176146>

32. Zhang D, Li G, Mu L, Thapa J, Li Y, Chen Z, Shi L, Su D, Son H, Pagan JA. Trends in medical school application and matriculation rates across the United States from 2001 to 2015: implications for health disparities. Acad Med 2021;96:885-893. <https://doi.org/10.1097/ACM.0000000000004033>
