# Supplementary material for: Socioeconomic and economic factors affecting access and progression in medical schools: a systematic review and meta-analysis
Source: J Educ Eval Health Prof. 2026 Apr 16;23:6. doi: 10.3352/jeehp.2026.23.6 (PMC13181141; doi:10.3352/jeehp.2026.23.6)
Supplement: Supplementary file 10 — Supplement 8. Supplementary analysis. [file jeehp-23-06-suppl8.docx]

**Supplement 8.** Supplementary analysis

To enable direct comparison of predictor effects on selection and progression, odds ratios were transformed into Cohen’s d using the formula:

$d=\frac{\sqrt{3}\times ln(OR)}{\pi}$ **Equation S1**

This transformation expresses the strength of association in standardized mean difference units. To summarize relative magnitudes, ratios of effects were calculated as follows:

1) Ratio 1= $\frac{Effect of parental income on progression}{Effect of parental income on selection}= \frac{-0.33}{-0.31}=1.06$

2) Ratio 2= $\frac{Effect of first-generation status on progression}{Effect of first-generation status on selection}= \frac{-0.30}{-0.26}=1.15$

3) Ratio 3= $\frac{Effect of household disadvantage on progression}{Effect of household disadvantage on selection}= \frac{-0.32}{-0.28}=1.14$

Ratios greater than 1 indicate that the predictor’s effect on progression was relatively stronger (i.e., more negative) than on selection.
